# Low LINC02147 expression promotes the malignant progression of oral submucous fibrosis

**DOI:** 10.1186/s12903-022-02346-4

**Published:** 2022-07-29

**Authors:** Jun Chen, Wenjie Li, Binjie Liu, Xiaoli Xie

**Affiliations:** 1 Hunan Key Laboratory of Oral Health Research & Hunan 3D, Printing Engineering Research Center of Oral Care and Hunan Clinical Research Center of Oral Major Diseases and Oral Health and Xiangya Stomatological Hospital and Xiangya School of Stomatology, Central South University, 72 Xiangya Road, Kaifu District, Changsha, 410008 People’s Republic of China; 2 State Key Laboratory of Powder Metallurgy, Central South University, Changsha, 410083 People’s Republic of China; 3 Department of Oral Health Science, School of Dentistry, University of Washington, Seattle, WA 98195 USA

**Keywords:** Oral submucous fibrosis (OSF), Oral squamous cell carcinoma (OSCC), lncRNA, Biomarker, Prognosis

## Abstract

**Background:**

Key lncRNAs associated with the malignant progression of oral submucous fibrosis (OSF) to oral squamous cell carcinoma (OSCC) were identified.

**Methods:**

Key lncRNAs with sequential changes from normal oral mucosa (NOM) to OSF to OSCC were identified based on the GEO database. Kaplan–Meier analysis was used to screen lncRNAs related to OSCC prognosis. Cox regression analysis was used to validate the independent prognostic value. qPCR was used to confirm the expression of the candidate lncRNAs. Gene set enrichment analysis (GSEA), nucleocytoplasmic separation assay, fluorescence in situ hybridization, RNA knockdown, western blot, and cell viability assay were performed to investigate the biological functions of the candidate lncRNA. A nomogram was constructed to quantitatively predict OSCC prognosis based on TCGA.

**Results:**

Bioinformatics methods indicated that LINC02147 was sequentially downregulated from NOM to OSF to OSCC, as confirmed by clinical tissues and cells. Meanwhile, low LINC02147 expression, as an independent prognostic factor, predicted a poor prognosis for OSCC. GSEA and in vitro studies suggested that low LINC02147 expression promoted OSF malignant progression by promoting cell proliferation and differentiation. A LINC02147 signature-based nomogram successfully quantified each indicator’s contribution to the overall survival of OSCC.

**Conclusions:**

Low LINC02147 expression promoted OSF malignant progression and predicted poor OSCC prognosis.

**Supplementary Information:**

The online version contains supplementary material available at 10.1186/s12903-022-02346-4.

## Background

Oral submucous fibrosis (OSF) is characterized by fibrosis of the oral mucosa. As a pre-cancerous lesion of oral squamous cell carcinoma (OSCC), OSF has a malignancy rate of 1.5–15% [1–6].

Epidemiologic studies have suggested that areca nut is a primary aetiologic factor responsible for OSF. Moreover, evidence has supported the role of genetic susceptibility and family history in the pathogenesis of OSF. With an increased number of areca nut chewers, OSF has shown gradual increases in incidence in recent years and has become a noticeable problem for global health [2]. Thus, identifying critical molecular events in the occurrence and progression of OSF will contribute to its early diagnosis and the development of targeted therapeutics.

Long noncoding RNAs (lncRNAs) exceed 200 nucleotides in length and act at the transcriptional and posttranscriptional levels to affect transcription, RNA processing, and translation. LncRNAs are essential in the pathogenesis of tumorigenesis, fibrosis, inflammation, and other diseases [7–9].

Studies have shown that lncRNAs play a role in OSF. LncRNA GAS5-AS1 is significantly downregulated in OSF tissues, and its overexpression significantly suppresses collagen contractility in arecoline-treated buccal mucosa fibroblasts (BMFs) [10]. LINC00974 promotes oral mucosa fibrogenesis by activating the TGF-β signaling pathway [11], while LINC00312 is upregulated in OSF specimens and positively correlated with fibrosis markers, including α-smooth muscle actin (α-SMA), collagen type 1, α1 (COL1α1), and fibronectin (FN1) [12]. The lncRNA hypoxia-inducible factor 1α-antisense RNA1 (HIF1A-AS1) is upregulated in OSF tissues and fibrotic BMFs. Moreover, arecoline increases HIF1A-AS1 expression in BMFs. Knockdown of HIF1A-AS1 suppresses the migration capacity of arecoline-treated BMFs [13]. Although these studies focused on lncRNAs in the pathogenesis of OSF, they did not investigate lncRNAs in the progression from OSF to OSCC.

To date, only two studies have investigated lncRNAs in the malignant progression of OSF to OSCC. Zhou et al. interpreted the lncRNA expression profile during the malignant evolution of normal oral mucosa (NOM)-OSF-OSCC at the genome-wide level and found 687 differentially expressed lncRNAs (DElncRNAs) during OSF progression, including 231 upregulated DElncRNAs and 456 downregulated DElncRNAs, indicating that lncRNAs were involved in different developmental stages of OSF [14]. Based on the RNA sequencing (RNA-seq) data, Zhou et al. found that the lncRNA ADAMTS9-AS2 was downregulated in OSCC tissues compared with OSF and NOM tissues. Low ADAMTS9-AS2 expression was associated with poor overall survival (OS) in OSCC. Exosome-derived ADAMTS9-AS2 suppressed the progression of OSF via the AKT pathway [15].

**Fig. 1:**
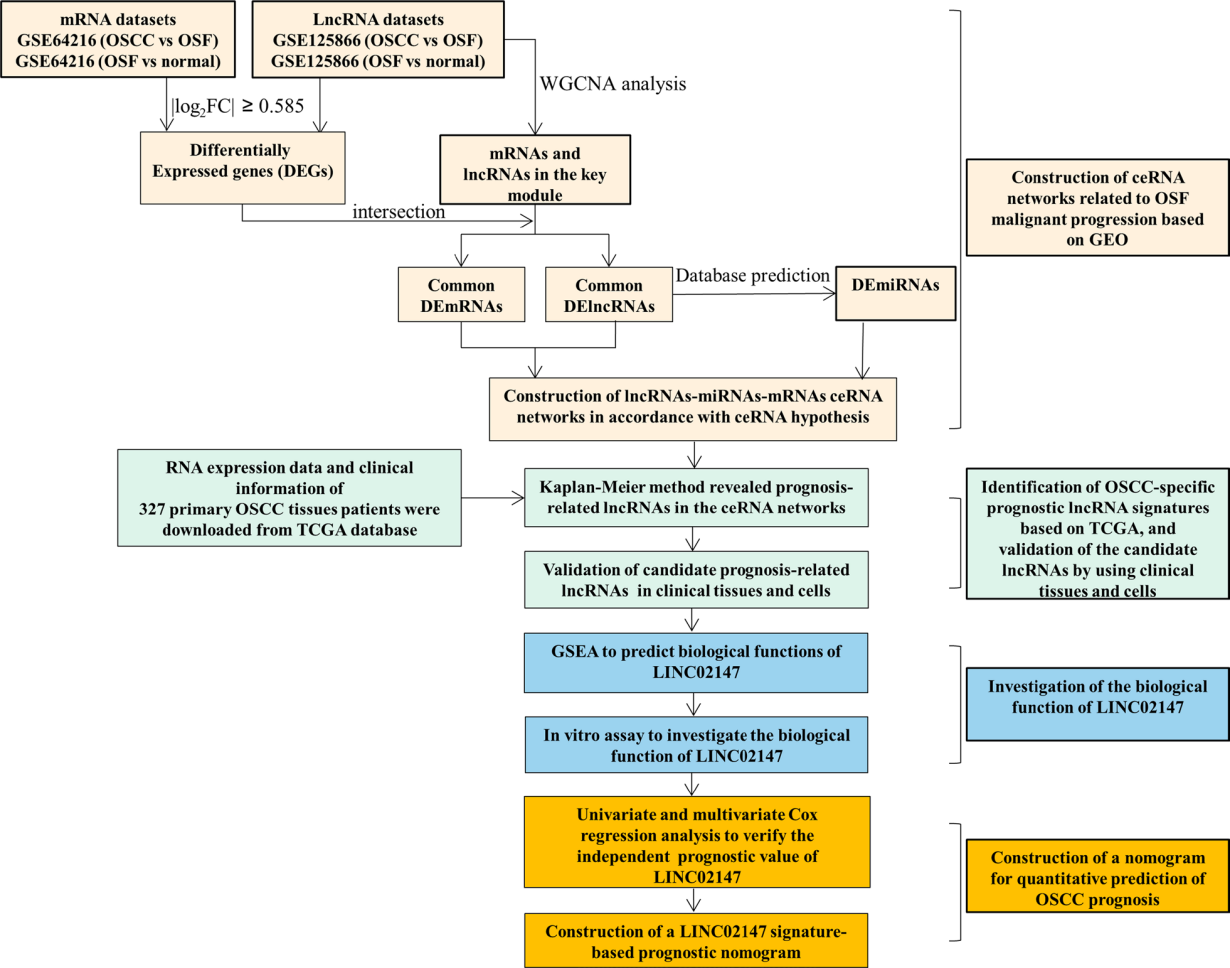
Workflow of this study. DEmRNAs, differentially expressed mRNAs; DElncRNAs, differentially expressed mRNAs; OSF, oral submucous fibrosis; OSCC, oral squamous cell carcinoma; GEO, Gene Expression Omnibus; TCGA, The Cancer Genome Atlas; WGCNA, weighted gene co-expression network analysis; GSEA, gene set enrichment analysis

This study aimed to identify key lncRNAs associated with OSF progression to OSCC and construct a novel nomogram for predicting OSCC prognosis. First, differentially expressed genes (DEGs) with consistently sequential changes from NOM to OSF to OSCC were identified based on Gene Expression Omnibus (GEO). Second, we constructed lncRNA-mediated ceRNA networks related to OSF progression. Third, lncRNAs with OSCC-specific prognostic characteristics were screened based on The Cancer Genome Atlas (TCGA). Then, the expression levels of candidate lncRNAs were validated in clinical tissues and cells. Finally, using bioinformatic methods, we identified 11 lncRNAs with a sequential change from NOM to OSF to OSCC based on ceRNA networks. A receiver operating characteristic (ROC) analysis and survival analysis among the 11 lncRNAs showed that LINC02147 has excellent diagnostic and prognostic value for OSCC. Its expression was also validated to be sequentially downregulated from NOM to OSF to OSCC in clinical tissues and cells. Gene set enrichment analysis (GSEA) and in vitro studies validated the biological function of LINC02147 in OSF malignant progression. A nomogram combining the LINC02147 signature and clinicopathologic factors was constructed to quantitatively predict OSCC prognosis. The workflow of this study is shown in Fig. 1.

## Materials & methods

### Identification of differentially expressed genes (DEGs)

#### Data collection

The raw data of the GSE125866 and GSE64216 datasets were downloaded from GEO (Additional file 1: Table S1). The whole gene list and samples were normalized for principal component analysis (PCA).

#### Data processing and differentially expressed gene analysis (DEGA)

The “*normalizeBetweenArrays*” in the “*limma*” package was used to read the microarray and normalize the expression data [16]. “*R*” package “*limma*”/edgeR further processed the expression files for DEGA between NOM and OSF, OSF and OSCC samples with biological replication. The cut-off criteria for screening DEGs were the *p*-value ≤ 0.05 and fold change (FC) ≥ 1.5 or ≤ 0.67.

#### Weighted gene co-expression network analysis (WGCNA)

WGCNA can divide genes into different modules through the biological network, helping to find important gene modules related to sample traits. WGCNA can complement the results of DEGA, make up for the deficiency of DEGA, and narrow the screening range of key genes.

The expression spectrum data of GSE125866 and the grouping information (NOM, OSF, and OSCC) were used as three traits for WGCNA. The “*WGCNA*” package in “*R*” was used to screen gene modules [17]. The correlation between modules and specific traits were analyzed. The modules with the strongest positive correlation and the strongest negative correlation were identified.

### Construction of ceRNA networks

The miRcode database website predicted the interactions of lncRNA-miRNA. The interactions of miRNA-mRNA were predicted by five database websites, including miRMap, miRanda, miRDB, TargetScan, and miTarBase. According to the ceRNA theory and Cytoscape V3.7, lncRNA-mediated ceRNA networks were constructed [18].

### Screening lncRNAs with prognostic and diagnostic values

#### Kaplan–Meier (K-M) survival analysis

The RNA-seq and clinical data of head and neck squamous cell carcinoma were downloaded from TCGA (http://tcgadata.nci.nih.gov/) to identify lncRNAs with OSCC-specific prognostic characteristics. Among which, 326 OSCC patients with no history of malignancy or neoadjuvant therapy were included in our study. The correlation between OS of OSCC patients and 11 lncRNAs in the ceRNA networks was analyzed by K-M methods. Details are provided in the Additional file 2: Methods.

#### ROC curve analysis

The “*pROC*” package was used to generate the ROC curve. The area under the ROC curve (AUC) was used as an accuracy index for evaluating the diagnostic performance of the candidate lncRNAs. The diagnostic accuracy based on the AUC value is defined as follows: 0.9–1.0, excellent; 0.8–0.9, good; 0.7–0.8, moderate; 0.6–0.7, fair; 0.5–0.6, poor. Generally, when AUC > 0.7, the candidate marker has a diagnostic value [19–21].

### Expression validation in clinical tissues and cells

#### Validation in clinical tissues

Fresh tissue samples of NOM, OSF, and OSCC were obtained from Xiangya Stomatological Hospital, Central South University. The study conformed to the Declaration of Helsinki and was approved by the Xiangya Stomatological Hospital ethics committee. All patients consented to the protocol approved by the institutional review board (Ethics Approval Number: 20200067). Exclusion criteria were those with any other history of oral lesions, drug treatment, or other systematic diseases. OSF was identified based on the 2005 World Health Organization classification system [22]. Ten NOM samples were obtained from healthy individuals without areca-chewing habits. Ten OSCC samples were obtained from areca-chewing patients. Ten OSF samples were collected 2 cm outside of the OSCC tissues and were confirmed pathologically with no OSCC tissues or neoplastic disease. All specimens were pathologically verified by three pathologists independently. The expression levels of LINC02147 and RP11-108K3.1 in NOM, OSF, and OSCC tissues were analyzed by quantitative PCR (qPCR). The primer sequences are listed in Additional file 1: Table S2.

#### Validation in cells

Primary hBMFs and OSF hBMFs were derived from histologically normal oral mucosa and OSF tissues, respectively (Ethics Approval Number: 20200067). Normal hBMFs and OSF hBMFs were cultured according to reported methods [11, 23]. OSCC cell line (SCC-9) was obtained from the American Type Culture Collection (Manassas, VA). Cells were cultured in DMEM (HyClone, USA) containing 15% or 10% fetal bovine serum (FBS; Gibco, USA). All cells were incubated at 37 °C in a humidified atmosphere of 5% CO_2_. The expression level of LINC02147 in cells was analyzed by qPCR.

### Prediction of LINC02147 biological function – GSEA

GSEA was used to predict functions and pathways of LINC02147 in the malignant progression of OSF. Genome-wide expression profiles in GSE125866 were used to rank all genes according to their correlations with LINC02147 expression. The ranking list was then used to calculate the enrichment score (ES) and *p*-value. Detailed steps of the procedure that appeared in the GSEA have been carried out in “*JAVA*” and “*R*”. We can download GSEA packages at www.broadinstitute.org/gsea/index.jsp. The canonical pathways gene sets (c2.cp.v4.0.symbols.gmt) from the Molecular Signatures Database (MsigDB) (http://www.broad.mit.edu/gsea/msigdb/index.jsp) were used for enrichment analysis. Gene sets represented by at least 15 genes were preserved [24, 25].

### In vitro study of LINC02147 in OSF malignant progression

#### Nucleocytoplasmic separation assay

The nucleocytoplasmic separation assay was performed to detect the subcellular location of LINC02147 in normal hBMFs. According to the protocol of PARIS™ Kit (Ambion, Austin, Tx., USA), total RNA can be partitioned into nuclear and cytoplasmic fractions. The isolated cytoplasm and nuclear RNA were used for subsequent qPCR. GAPDH and U6 were used as internal references for RNA from the cytoplasm and nuclear, respectively.

#### RNA fluorescence in situ hybridization (FISH)

RNA FISH assay further determined the subcellular location of LINC02147 in normal hBMFs. Cy3 fluorescein-labeled probes against U6 snRNA and LINC02147 were designed and synthesized by RIBOBIO (Guangzhou, China). The FISH assay was conducted according to the manufacturer’s protocol of the Fluorescence in Situ Hybridization Kit (RIBOBIO Biotechnology, Guangzhou, China). Nuclei were counterstained with DAPI. Fluorescence signals were scanned by using an inverted fluorescence microscope (Nikon, Tokyo, Japan).

#### RNA knockdown

Ribo™ lncRNA Smart Silencer for LINC02147 was designed and purchased from Guangzhou RIBOBIO (Guangzhou, China). The product contains a mixture of six target sequences for LINC02147, including 5’-GTCCTCACGTGGCCTCTTT-3’, 5’-CAAGATCAAGGTGCTATCA-3’, 5’-CTGGCTTGTAGACAGCTAT-3’, 5’-CAGGGTTGGTTTCGGCTGTG-3’, 5’-CAAGATCAAGGTGCTATCAG-3’, 5’-TCCTCACGTGGCCTCTTTGT-3’. The Ribo FECT CP Transfection Kit was used to transfect LINC02147-siRNA or negative control (NC)-siRNA into normal hBMFs and SCC-9.

#### qPCR

The expression levels of LINC02147, α-SMA, COL1α1, FN1, vimentin, MCM2, MCM3, and MCM5 were examined by qPCR. Details of the qPCR assay are provided in the Additional file 2: Methods. The primer sequences used in qPCR are listed in Additional file 1: Table S2.

#### Western blot (WB)

The expression levels of α-SMA, COL1α1, FN1, and vimentin were examined by WB. The following antibodies were used: anti-α-SMA (Abcam, ab124964), anti-COL1α1 (Abcam, ab260043), anti-FN1 (Abcam, ab45688), anti-vimentin (Abcam, ab92547) and anti-β-actin (Immunoway, YM3028). Details are provided in the Additional file 2: Methods*.*

#### Cell viability assay

Cell Counting Kit-8 (CCK-8; Dojindo, Japan) was used to detect cell viability according to the manufacturer’s guidance. Normal hBMFs were incubated at 37 °C for 0 h, 24 h, 48 h, and 72 h. SCC-9 cells were incubated at 37 °C for 0 h, 12 h, 24 h, and 48 h. An enzyme-labeled instrument (BioTeck, Epoch, USA) was used to determine the cell viability by the absorbance at 490 nm or 450 nm. The experiments were repeated three times.

### Independent prognostic value analysis

Cox proportional hazards models were used to estimate hazard ratios (HR) to validate the prognostic value of LINC02147 further and identify independent prognostic factors. The clinicopathological characteristics of the 326 OSCC patients from TCGA are shown in Additional file 1: Table S3. Nine characteristics were selected for univariate Cox regression analysis. Then, the characteristics with statistical significance in univariate Cox regression analysis were selected for multivariate Cox regression analysis to identify independent prognostic factors. Details are provided in the Additional file 2: Methods. When HR > 1, the characteristic is considered a risk factor. When HR < 1, the characteristic is considered a protective factor [26].

### Construction and validation of a predictive nomogram

Based on the independent prognostic factors screened out by multivariate Cox regression analysis, a nomogram was constructed using the “*rms*” package in “*R*” (version 4.0). The nomogram was used to predict the OS rate for OSCC quantitatively. The calibration plots evaluated the consistency between actual OS and predicted OS created by the constructed nomogram. The concordance index (C-index), ranging from 0.5 to 1.0 (0.5 indicates completely random, 1 indicates entirely consistent), was used to determine the predictive accuracy of the nomogram [27]. The “*survConcordance*” in the “*survival*” package was used to calculate C-index.

### Statistical analysis

All statistical analyses were performed using SPSS 20.0 software (SPSS Inc., USA) or GraphPad Prism 8 (La Jolla, USA). Student’s t-test and Wilcoxon test were used for analyzing two-group comparisons. One-way ANOVA was used for the comparison of multiple groups. The K-M method, log-rank test, and Cox regression analysis were performed to evaluate survival outcomes. Two-way ANOVA was used for CCK-8 data analysis. All results were expressed as the mean value ± standard deviation (SD) for at least three separate experiments. Differences were considered statistically significant at *p* < 0.05.

## Results

### Construction of ceRNA networks related to the malignant progression of OSF

We obtained 271 DEmRNAs (93 upregulated and 178 downregulated) and 21 DElncRNAs (8 upregulated and 13 downregulated) with sequential changes from NOM to OSF to OSCC using DEGA. Details are provided in the Additional file 3: Results.

The “brown” module had 13,993 upregulated genes, including mRNAs and lncRNAs, based on the WGCNA. The “orangered4 + plum1” modules had 6186 downregulated genes, including mRNAs and lncRNAs. Details are provided in the Additional file 3: Results.

**Fig. 2:**
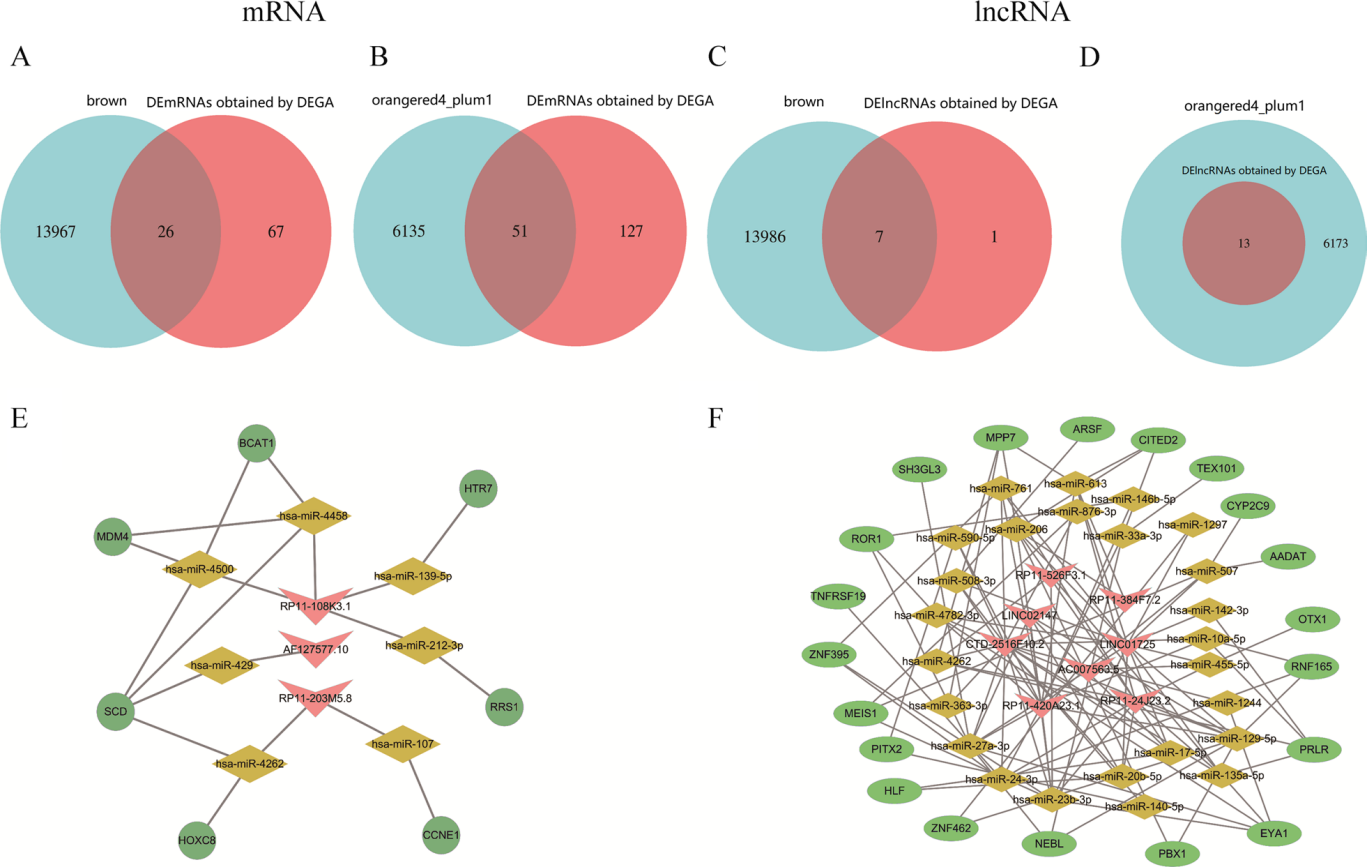
Construction of ceRNA network related to malignant progression of OSF. **A** The red area represents the number of upregulated DEmRNAs obtained by differentially expressed genes analysis (DEGA). The blue area represents the number of the most significant positive genes analyzed by WGCNA. The overlapping area represents the number of common upregulated DEmRNAs obtained by the two methods. **B** The red area represents the number of downregulated DEmRNAs obtained by DEGA. The blue area represents the number of the most significant negative genes analyzed by WGCNA. The overlapping area represents the number of common downregulated DEmRNAs obtained by the two methods. **C** The red area represents the number of upregulated DElncRNAs obtained by DEGA. The blue area represents the number of the most significant positive genes analyzed by WGCNA. The overlapping area represents the number of common upregulated DElncRNAs obtained by the two methods. **D** The red area represents the number of downregulated DElncRNAs obtained by DEGA. The blue area represents the number of the most significant negative genes analyzed by WGCNA. The overlapping area represents the number of common downregulated DElncRNAs obtained by the two methods. **E** The diagram of upregulated lncRNA- miRNA- upregulated mRNA network. **F** The diagram of downregulated lncRNA- miRNA- downregulated mRNA network

The WGCNA and DEGA results were crossed to narrow the screening range of key genes. Finally, 26 upregulated mRNAs (Fig. 2A), 51 downregulated mRNAs (Fig. 2B), 7 upregulated lncRNAs (Fig. 2C), and 13 downregulated lncRNAs (Fig. 2D) with sequential changes from NOM to OSF to OSCC were identified to construct ceRNA networks. The upregulated-lncRNA-mediated ceRNA network contains 3 lncRNAs, 7 miRNAs, and 7 mRNAs (Fig. 2E). The downregulated-lncRNA-mediated ceRNA network contains 8 lncRNAs, 25 miRNAs, and 20 mRNAs (Fig. 2F).

Ultimately, 11 lncRNAs that may play a role in OSF malignant progression were identified based on the ceRNA networks (Additional file 1: Table S4).

### LINC02147 and RP11-108K3.1 showed promising prognostic and diagnostic potential for OSCC

**Fig. 3:**
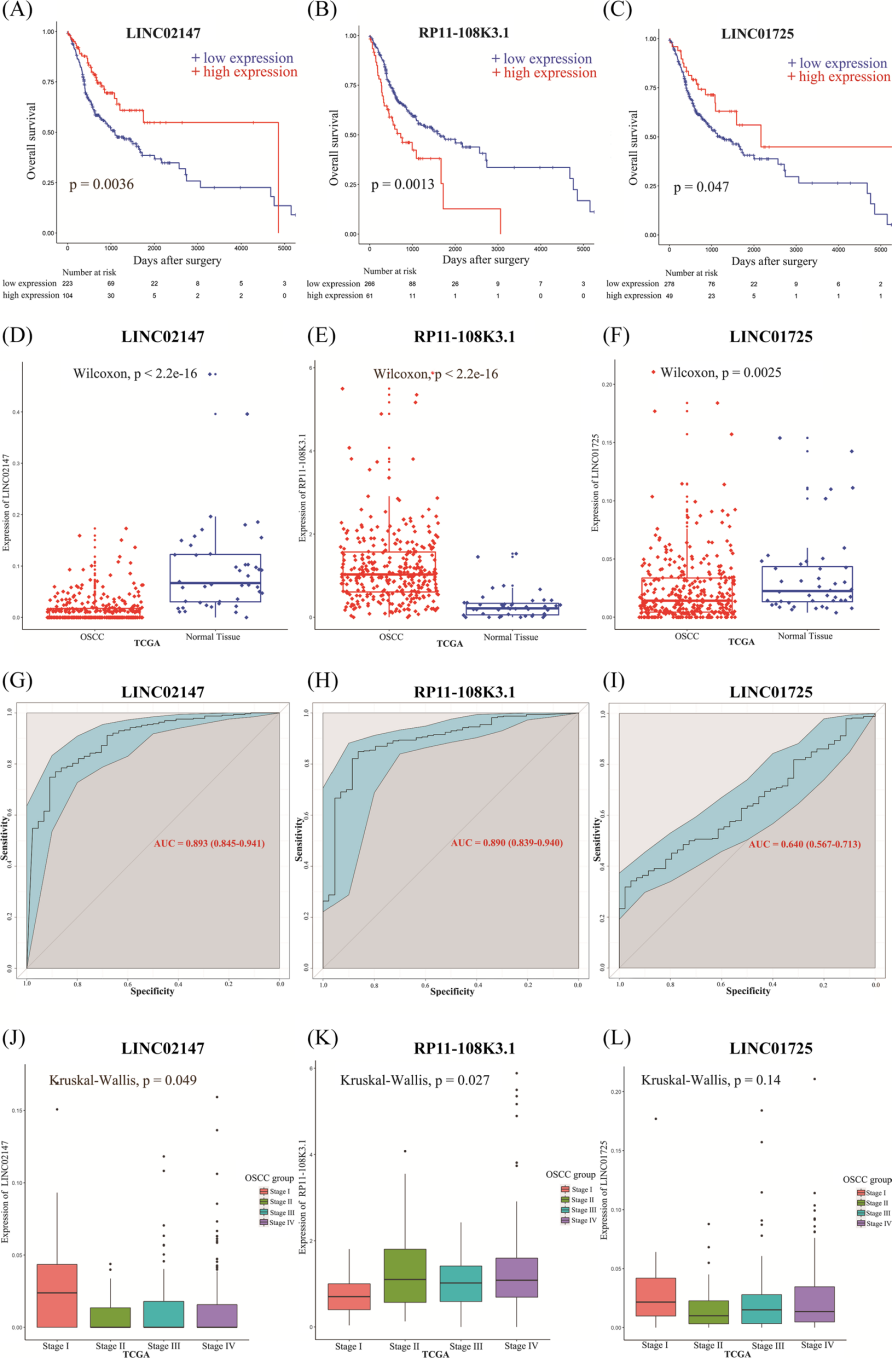
Kaplan–Meier (K–M) survival analysis and ROC analysis of lncRNA signatures from TCGA OSCC dataset. **A** The K–M survival curve showed that low LINC02147 expression resulted in worse overall survival (OS) in OSCC patients. **B** The K–M survival curve showed that high expression of RP11-108K3.1 resulted in worse OS in OSCC patients. **C** The K–M survival curve showed that low expression of LINC01725 resulted in worse OS in OSCC patients. **D**–**F** Validation of LINC02147, RP11-108K3.1, and LINC01725 expression in TCGA. **G** The ROC curve showed the AUC of LINC02147 was 0.893. **H** The ROC curve showed the AUC of RP11-108K3.1 was 0.890. **I** The ROC curve showed the AUC of LINC01725 was 0.640. **J**–**L** Comparison of expression levels for LINC02147, RP-108K3.1, and LINC01725 in OSCC of different TNM stages. Global differences in survival curves were compared by log-rank test. Expression differences in OSCC and normal tissues were compared by the Wilcoxon test. Expression differences in OSCC of different TNM stages were compared by the Kruskal–Wallis test

Among the 11 lncRNAs in the ceRNA networks, only 3 lncRNAs (LINC02147, RP11-108K3.1, and LINC01725) were associated with OS in OSCC patients. OSCC patients with low LINC02147 expression and LINC01725 had significantly poorer OS than those with high expression (Fig. 3A–C). OSCC patients with high expression of RP11-108K3.1 had poorer OS than those with low expression (Fig. 3B). Meanwhile, the expression levels of the 3 lncRNAs were validated in TCGA. Compared with normal samples, LINC02147 and LINC01725 were significantly downregulated in the OSCC samples, whereas RP11-108K3.1 was significantly upregulated (Fig. 3D–F).

Furthermore, the diagnostic values of the 3 lncRNAs were evaluated. LINC02147 (AUC = 0.893) and RP11-108K3.1 (AUC = 0.890) were able to distinguish OSCC from normal controls (Fig. 3G, H), but LINC01725 (AUC = 0.640) did not show good diagnostic performance (Fig. 3I). In addition, the expression levels of the 3 lncRNAs in different clinical stages of OSCC were assessed. The expression level of LINC02147 in the stage I OSCC samples was significantly higher than that in the stage II, III, and IV samples (Fig. 3J). The expression level of RP11-108K3.1 in the stage I OSCC samples was significantly lower than that in the stage II, III, and IV samples (Fig. 3K). The expression level of LINC01725 in the stage I OSCC samples was higher than that in the stage II, III, and IV samples, but the difference was not statistically significant (*p* = 0.14) (Fig. 3L). Therefore, these results suggested that LINC02147 and RP11-108K3.1 might be potential markers for the early diagnosis of OSCC while LINC01725 is not.

Since LINC02147 and RP11-108K3.1 showed good prognostic and diagnostic values, we subsequently validated the expression of these two genes in clinical tissue samples.

### LINC02147 was sequentially downregulated from NOM to OSF to OSCC in clinical tissues and cells

**Fig. 4:**
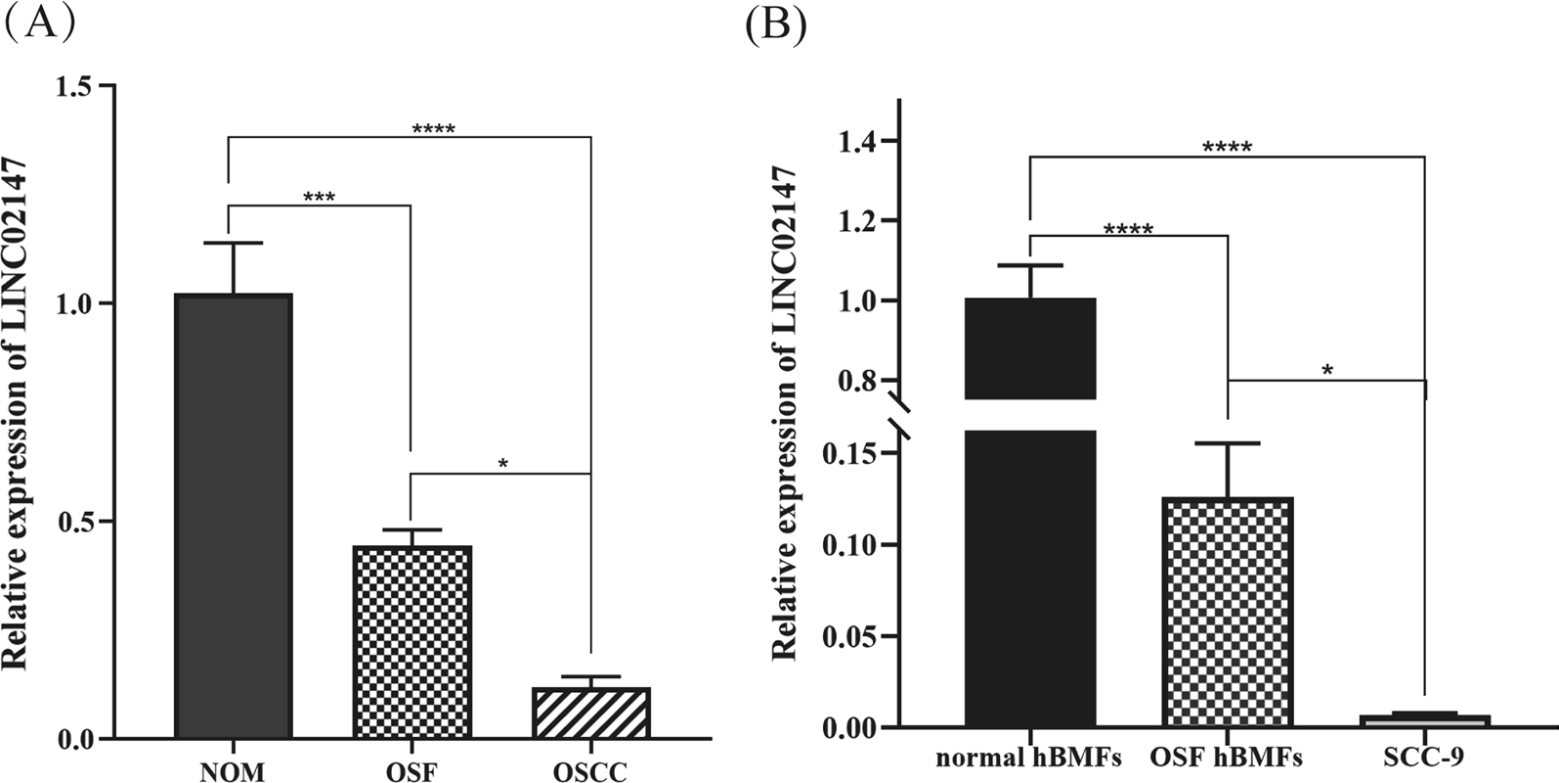
Validation of LINC02147 expression in clinical tissues and cells. **A** The relative expression of LINC02147 was subsequentially downregulated from NOM to OSF to OSCC clinical tissues. **B** The relative expression of LINC02147 was subsequentially downregulated from normal hBMFs to OSF hBMFs to SCC-9 cells. Expression differences were compared by ordinary one-way ANOVA test (*p < 0.05, **p < 0.01, ***p < 0.001, ****p < 0.0001)

The expression levels of LINC02147 and RP11-108K3.1 were measured by qPCR in clinical tissues and cells. LINC02147 expression was sequentially downregulated from NOM to OSF to OSCC (Fig. 4A), which is consistent with the bioinformatics results. Moreover, LINC02147 expression was sequentially downregulated from normal hBMFs to OSF hBMFs to SCC-9 cells (Fig. 4B). However, the expression of RP11-108K3.1 was significantly downregulated in OSF and OSCC (*p* < 0.05) (Additional file 4: Fig. S1), which was inconsistent with the bioinformatics results. Therefore, only LINC02147 was verified in clinical tissues and cells, while RP11-108K3.1 was not; therefore, we chose LINC02147 for further studies.

### GSEA predicted that LINC02147 was involved in OSF malignant progression by negatively regulating proliferation-related biological processes and the MCM pathway

**Fig. 5:**
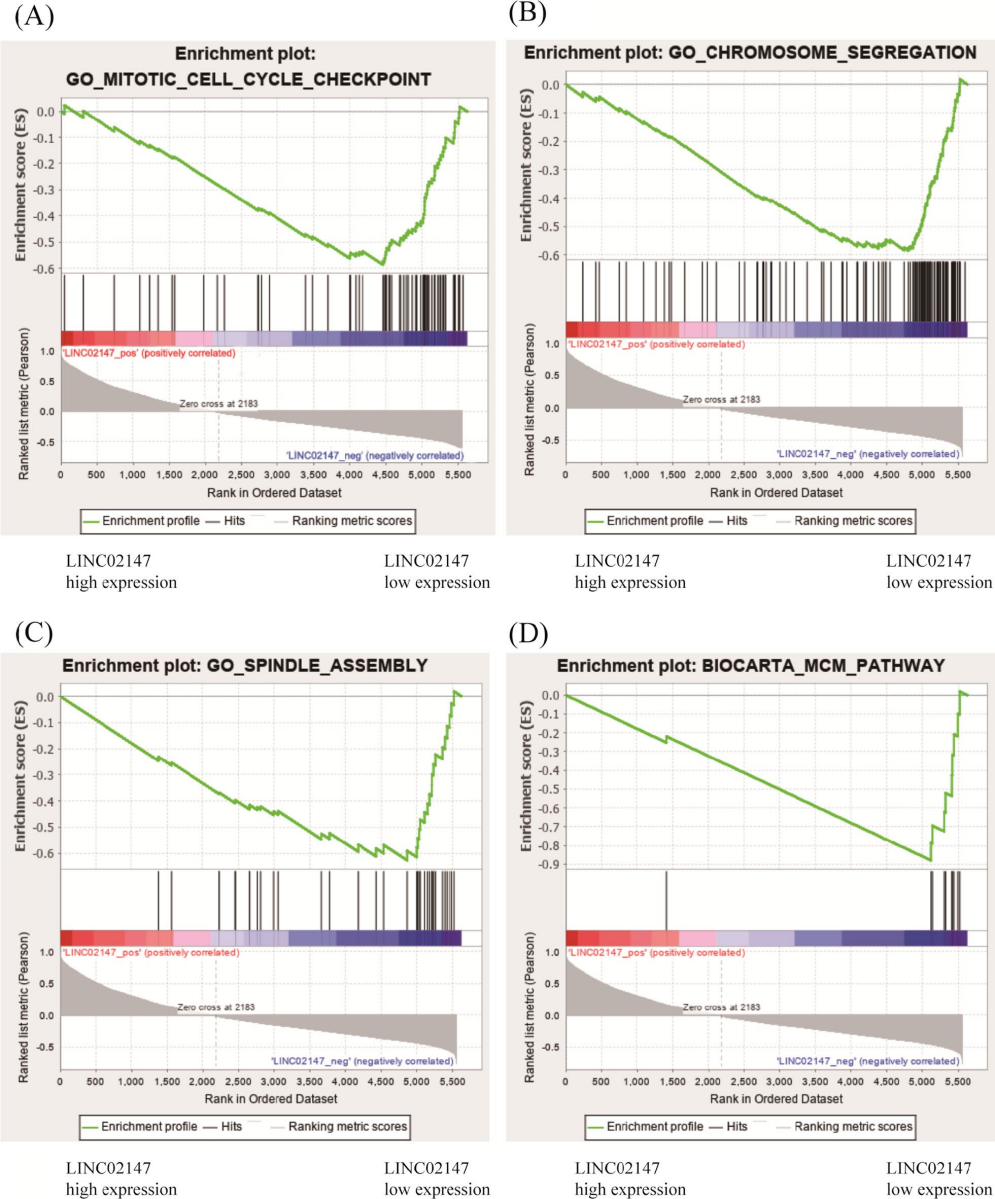
Prediction of LINC02147 biological function by GSEA. (**A**–**C**) Function enrichment plots of GSEA showed gene signatures of mitotic cell cycle checkpoint, chromosome segregation, and spindle assemble in patients with lower LINC02147 expression were more active than in patients with higher LINC02147 expression. **D** Pathway enrichment plots of GSEA showed gene signatures of minichromosome maintenance (MCM) pathway in patients with lower LINC02147 expression were more active than in patients with higher LINC02147 expression. The enrichment score (ES, green line) means the degree to which the gene set is overrepresented at the top or bottom of the ranked list of genes

Functional enrichment plots of GSEA showed that gene signatures of mitotic cell cycle checkpoint, chromosome segregation, and spindle assemble in patients with low LINC02147 expression were more active than in patients with high LINC02147 expression, indicating that LINC02147 was negatively correlated with these three biological processes (Fig. 5A–C).

Pathway enrichment plots of GSEA showed that gene signatures of the minichromosome maintenance (MCM) pathway were more active in patients with low LINC02147 expression than in patients with high LINC02147 expression, indicating that LINC02147 was negatively correlated with the MCM pathway (Fig. 5D).

### In vitro study showing how low LINC02147 expression promoted the malignant progression of OSF

#### Knockdown of LINC02147 promoted fibrogenesis in hBMFs

**Fig. 6:**
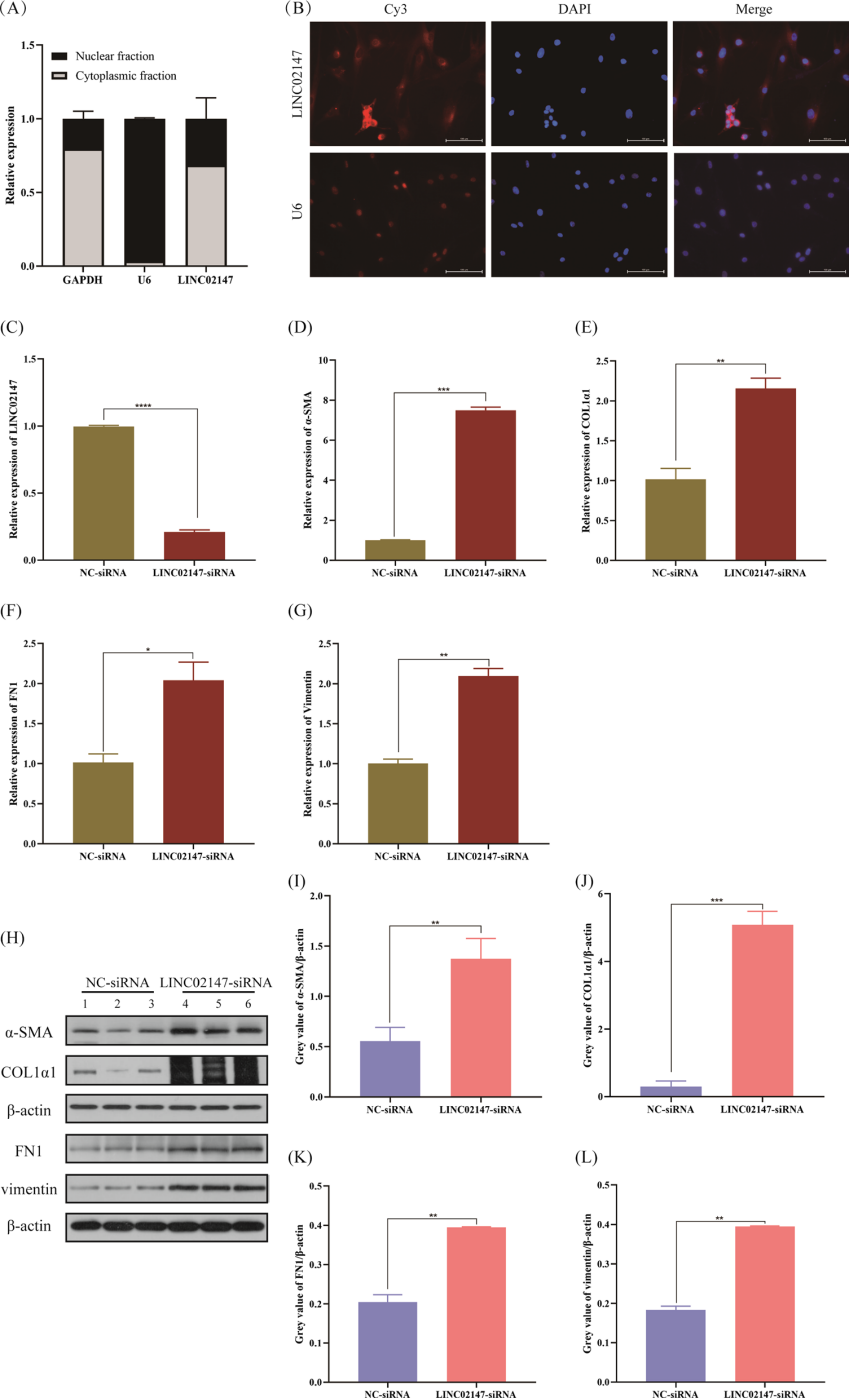
Knockdown of LINC02147 promoted fibrogenesis in hBMFs. **A** The expression level of LINC02147 in the subcellular fractions of hBMFs was detected by qPCR. GAPDH and U6 were used as cytoplasmic and nuclear markers, respectively. **B** RNA FISH assay was used to determine the subcellular location of LINC02147 in hBMFs. Nuclei were stained with DAPI. Scale bar = 10 μm. **C**–**G** qPCR analysis of LINC02147, α-SMA, COL1α1, fibronectin (FN1), and vimentin in hBMFs with NC siRNA or LINC02147 siRNA. **H**–**L** Western blot results of α-SMA, COL1α1, FN1, and vimentin in hBMFs with NC siRNA or LINC02147 siRNA. The blots images were cropped by ImageJ (version1.8.0.112), and full-length blots are presented in Additional files 5 and 6: Figs. S2, S3. Unpaired t-test was used to compare gene expression between two groups (*p < 0.05, **p < 0.01, ***p < 0.001, ****p < 0.0001)

The nucleocytoplasmic separation assay and FISH assay showed that LINC02147 was mainly located in the cytoplasm in hBMFs (Fig. 6A, B).

The results showed that knockdown of LINC02147 significantly elevated the expression levels of α-SMA, COL1α1, FN1, and vimentin at both the RNA and protein levels in hBMFs (Fig. 6C–L).

#### Knockdown of LINC02147 promoted the cell proliferation of hBMFs

**Fig. 7:**
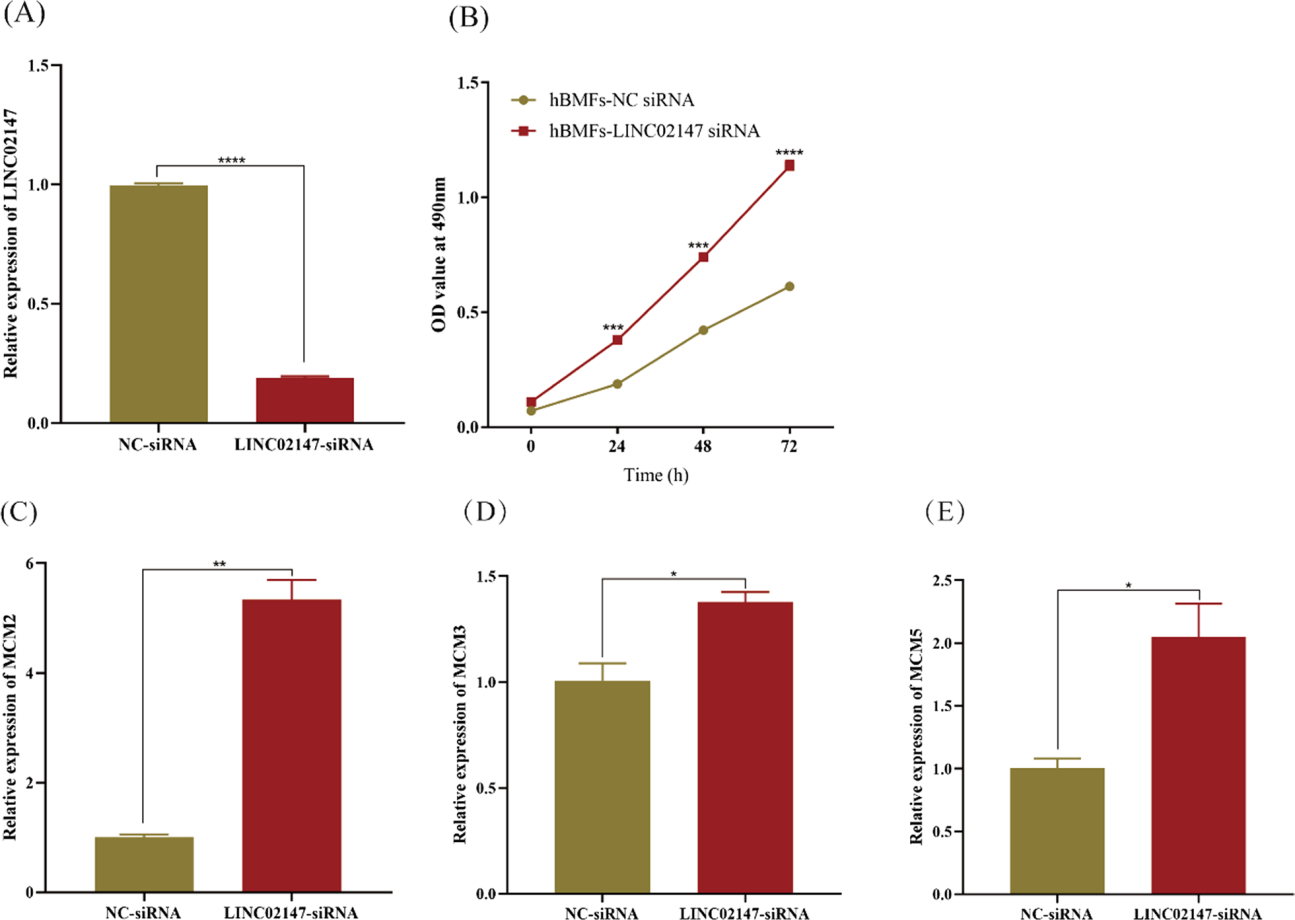
Knockdown of LINC02147 promoted cell proliferation of hBMFs. **A**–**B** Knockdown of LINC02147 promoted cell proliferation of SCC-9. **C**–**E** qPCR analysis of MCM2, MCM3, and MCM5 in hBMFs with NC siRNA or LINC02147siRNA. Two-way ANOVA was used for CCK-8 data analysis. Unpaired t-test was used to compare gene expression between two groups (*p < 0.05, **p < 0.01, ***p < 0.001, ****p < 0.0001)

CCK-8 assay showed that knockdown of LINC02147 promoted the cell proliferation of hBMFs (Fig. 7A, B). GSEA predicted that LINC02147 was involved in OSF malignant progression by negatively regulating the MCM pathway. MCM2, MCM3, and MCM5 are major molecules in the MCM pathway. They are not only specific biomarkers of cell proliferation [28] but also potential biomarkers for OSCC [29–32]. Our in vitro study showed that knockdown of LINC02147 significantly elevated the expression levels of MCM2, MCM3, and MCM5 in hBMFs (Fig. 7C–E).

#### Knockdown of LINC02147 promoted SCC-9 cell proliferation

**Fig. 8:**
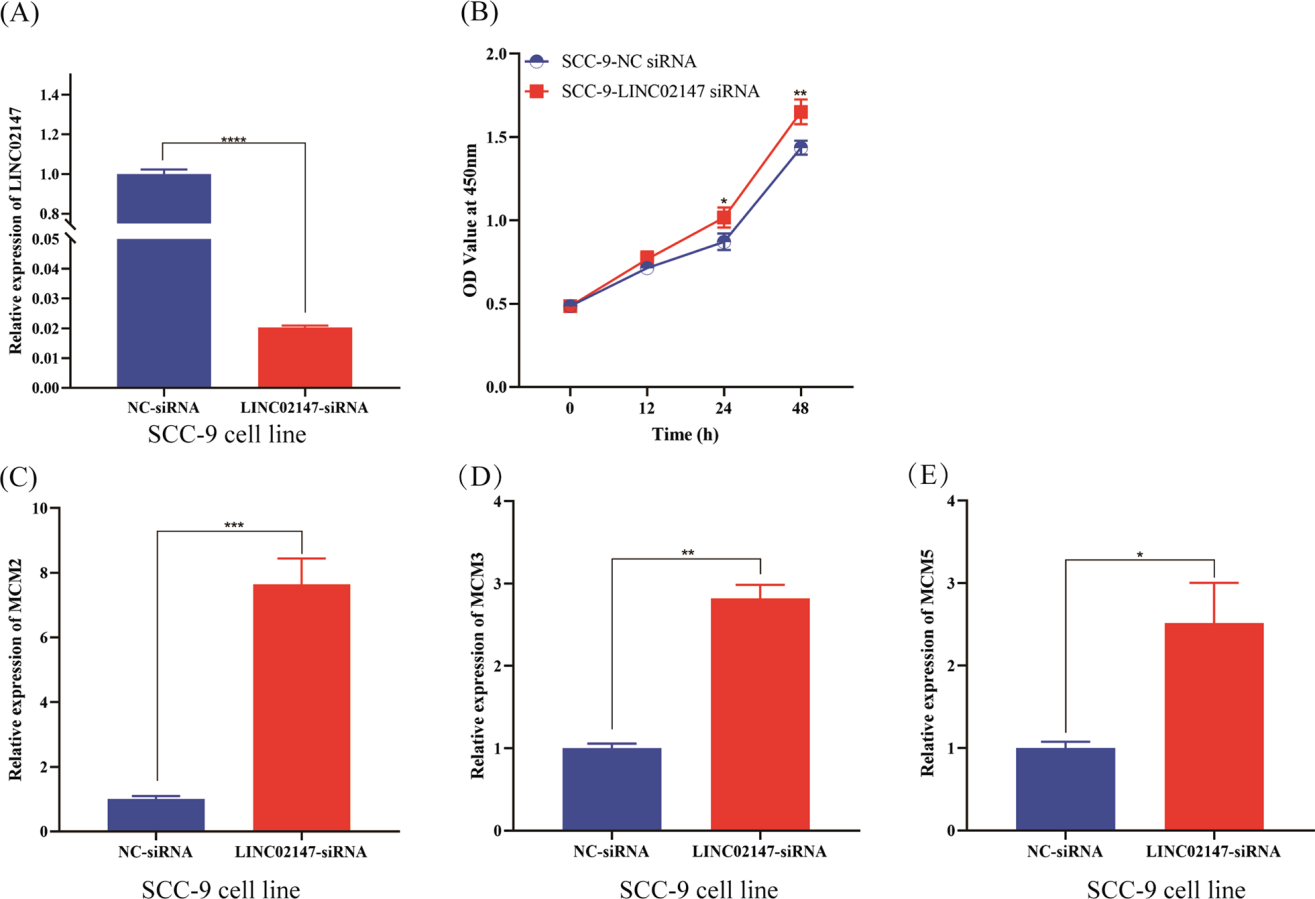
Knockdown of LINC02147 promoted cell proliferation of SCC-9. **A**, **B** Knockdown of LINC02147 promoted cell proliferation of SCC-9. **C**–**E** qPCR analysis of MCM2, MCM3, and MCM5 in SCC-9 cells with NC siRNA or LINC02147siRNA. Two-way ANOVA was used for CCK-8 data analysis. Unpaired t-test was used to compare gene expression between two groups (*p < 0.05, **p < 0.01, ***p < 0.001, ****p < 0.0001)

CCK-8 assay showed that knockdown of LINC02147 promoted the proliferation of SCC-9 cells (Fig. 8A, B). Knockdown of LINC02147 increased the expression of MCM2, MCM3, and MCM5 in SCC-9 cells (Fig. 8C–E).

### Low LINC02147 expression was independently associated with a poor prognosis of OSCC

**Fig. 9:**
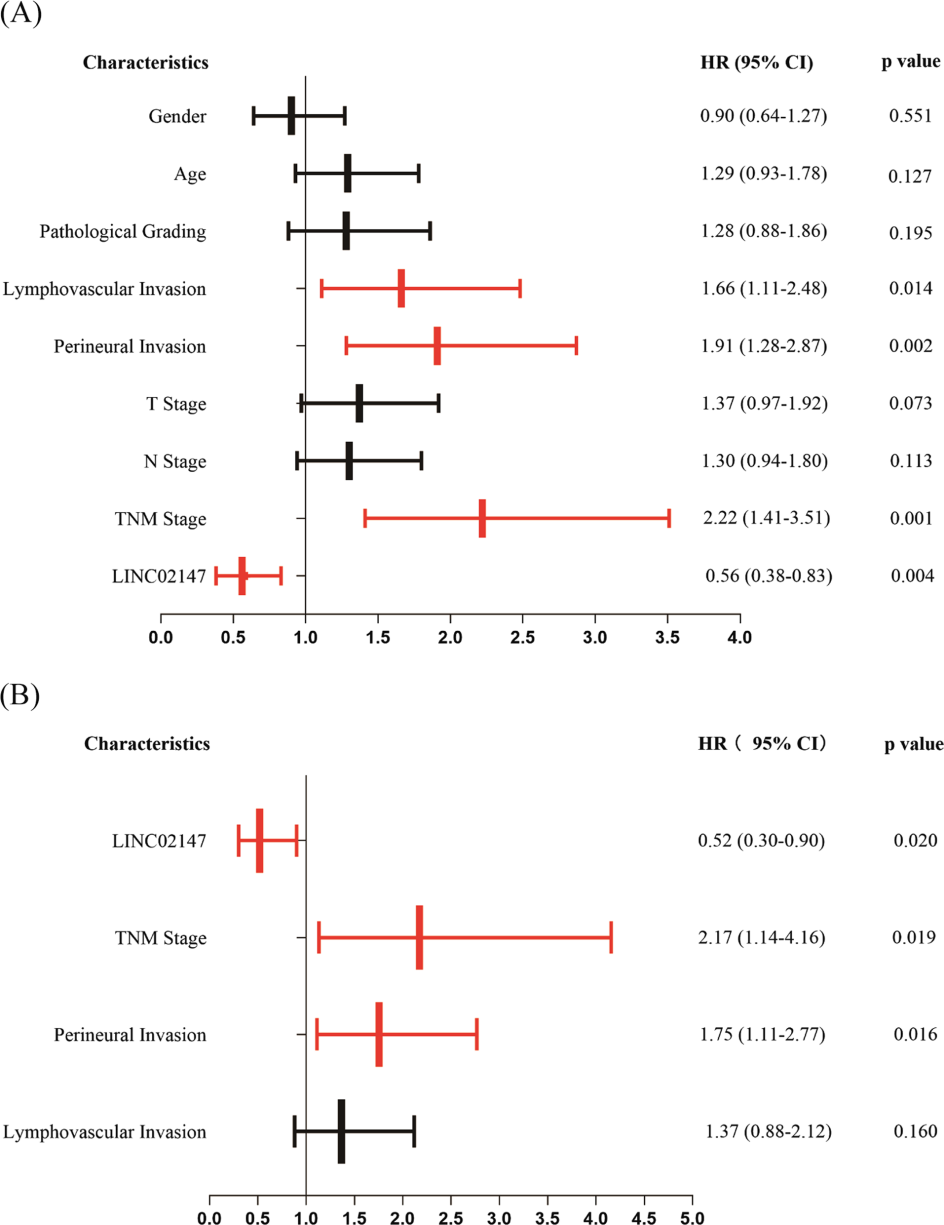
Univariate and multivariate Cox proportional hazards regression analysis. **A** The hazard ratio (HR) and p values of the 9 characteristics were calculated by univariate Cox regression. **B** The HR and p values of the 4 characteristics were calculated by multivariate Cox regression

Univariate Cox regression analysis showed that the status of LINC02147, TNM stage, lymphovascular invasion and perineural invasion were related to the OS of OSCC patients. The HR of LINC02147 was 0.56 (95% CI = 0.38–0.83; *p* = 0.004), indicating that low LINC02147 expression predicted poorer OS in OSCC patients. TNM stage (HR = 2.22, 95% CI = 1.41–3.51, *p* = 0.001), lymphovascular invasion (HR = 1.66, 95% CI = 1.11–2.48, *p* = 0.014), and perineural invasion (HR = 1.91, 95% CI = 1.28–2.87, *p* = 0.002) were risk factors for OSCC (Fig. 9A).

Multivariate Cox regression analysis showed that LINC02147, TNM stage, and perineural invasion were all independently related to the OS of OSCC, indicating that low LINC02147 expression was independently associated with poor prognosis of OSCC (HR = 0.52, 95% CI = 0.30–0.90, *p* = 0.020) (Fig. 9B).

### LINC02147 signature-based nomogram for the quantitative prediction of OSCC prognosis

**Fig. 10:**
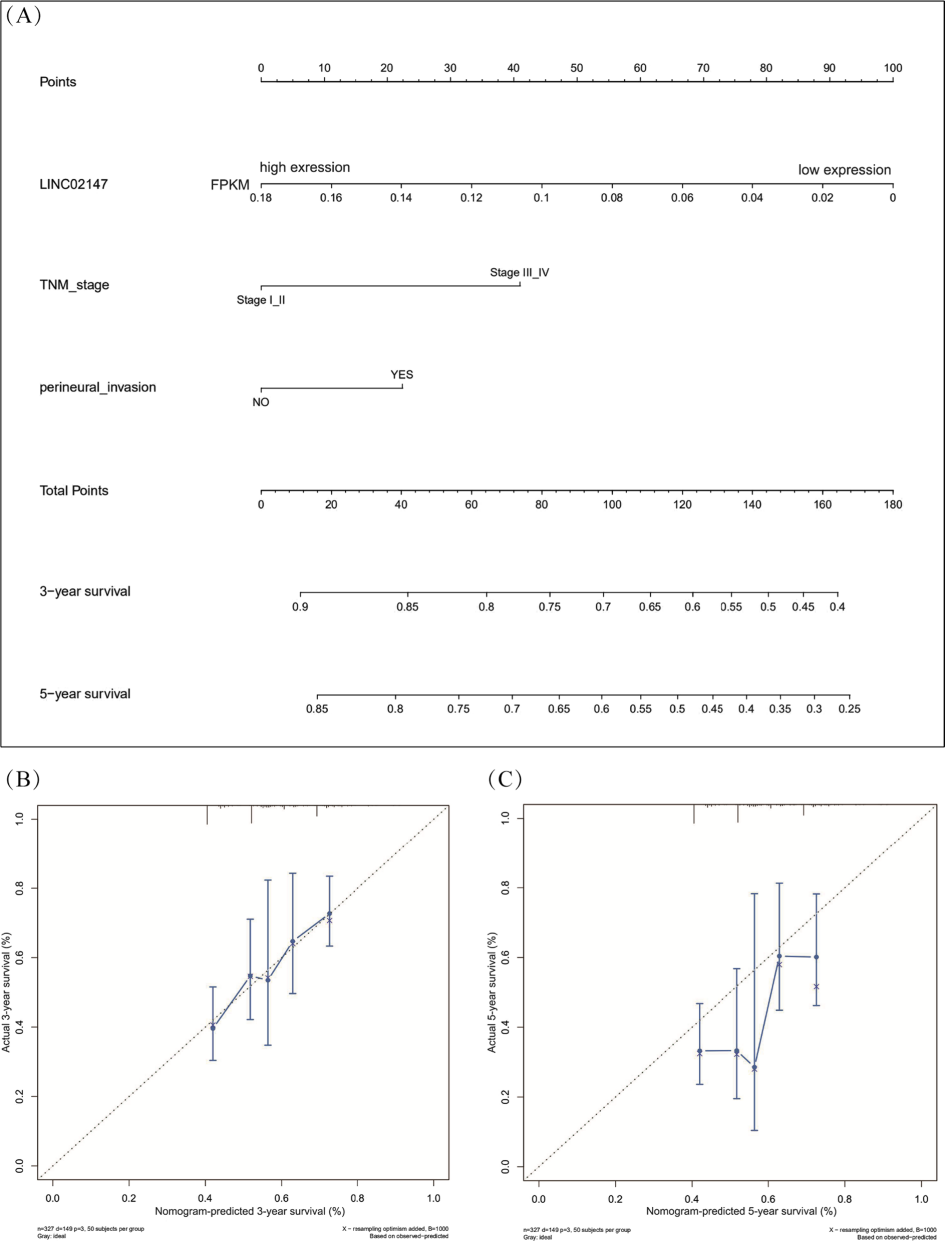
Construction and validation of LINC02147 signature-based nomogram. **A** The nomogram integrates three independent prognostic factors: LINC02147, TNM stage, and perineural invasion, predicting 3-year and 5-year survival for OSCC patients. Each factor corresponds to a point value in the top row, indicating its contribution to the overall survival (OS) of OSCC patients. Add these values together to get total points. Then, a line was drawn down from the “total points” to the survival axis to determine the probability of survival at 3-year or 5-year. **B**, **C** Calibration curves showed the nomogram predicted survival matched actual survival well at 3-year and 5- year

A nomogram was constructed to quantitatively predict OS based on the 3 independent prognostic factors (LINC02147 signature, TNM stage, and perineural invasion). Points in the nomogram were assigned to represent the contribution of each factor to OS. Low LINC02147 expression accounted for 100 points, indicating that the LINC02147 signature was a vital OS predictor for OSCC (Fig. 10A). Calibration curves showed that the predictive OS matched well with the actual OS, especially at 3 year (Fig. 10B, C). The C-index of the nomogram was 0.624 (95% CI = 0.577 ~ 0.670, *p* = 3e-04), indicating that the nomogram had good accuracy and sensitivity.

## Discussion

The mechanism of OSF malignant progression remains unclear. Previous studies have suggested that arecoline-induced inactivation of tumour suppressor genes [33], DNA damage [34, 35], hypoxia [36], and fibroblast senescence [37] contribute to OSF malignant progression.

Studies have demonstrated that lncRNAs are essential in the pathogenesis of fibrosis [8] and tumorigenesis [7]. However, studies of lncRNAs in the occurrence and malignant progression of OSF are still in nascent. To date, only 8 lncRNAs have been found to be related to the pathogenesis of OSF [38], including H19 [39], LINC00084 [40], HIF1A-AS1 [13], LINC00312 [12], LINC00974 [11], HOTTIP [41], GAS5-AS1 [10] and ADAMTS9-AS2 [15]. These lncRNAs are involved in the pathogenesis of OSF mainly by regulating myofibroblast activation [10–13, 39–41] and epithelial to mesenchymal transition (EMT) [40]. In addition, only ADAMTS9-AS2 has been confirmed to be associated with the malignant progression of OSF to OSCC [15]. Our study identified the involvement of a novel lncRNA, LINC02147, in the occurrence and malignant progression of OSF.

In this study, we constructed lncRNA-related ceRNA networks associated with the malignant progression of OSF. The potential biological functions and pathways of DEGs in the ceRNA networks are provided in the Additional file 3: Results. Based on the ceRNA networks, 11 lncRNAs with a sequential change from NOM to OSF to OSCC were identified (Additional file 1: Table S4). Among the 11 lncRNAs, LINC02147 has excellent diagnostic and prognostic value for OSCC, and its expression was also validated in clinical tissues and cells (Fig. 4). LINC02147, also named CTD-3179P9.1 (Ensembl ID: ENSG00000249797), is located on 5q23.1. Zhou et al. also found that LINC02147 was sequentially downregulated from NOM to OSF to OSCC [14], but they only studied the expression of LINC02147. To the best of our knowledge, our study is the first to investigate the biological function of LINC02147.

Confirming the subcellular localization of LINC02147 will aid in studying its biological function. The nucleocytoplasmic separation assay and RNA FISH assay showed that LINC02147 was mainly located in the cytoplasm of hBMFs. This result was consistent with a previous study, which predicted the subcellular location of LINC02147 in the cytoplasm using the lncLocator website [42]. Our study is the first to identify the subcellular localization of LINC02147 by cell assay, which will provide a reference for further studies on the mechanism of LINC02147.

GSEA predicted that LINC02147 was involved in OSF malignant progression by negatively regulating the mitotic cell cycle checkpoint, chromosome segregation, spindle assemble, and MCM pathway (Fig. 5). Mitotic cell cycle checkpoint, chromosome segregation, and spindle assemble are essential processes of cell proliferation and cell differentiation, the abnormal regulation of which could lead to malignant progression [43–46]. The MCM family plays a central role in DNA replication [28]. MCMs are considered specific biomarkers of cell proliferation because MCMs are highly expressed in proliferating cells but have no or poor expression in stationary or well-differentiated cells [28]. Studies have shown that MCM2, a member of the MCM family, is overexpressed in OSCC and serves as an effective biomarker for OSCC [29, 30]. A previous transcriptome analysis suggested MCM2 as a pan-cancer biomarker [47]. Some studies have found that MCM3 and MCM5 are potential biomarkers for OSCC [31, 32]. High expression levels of MCM5 may serve as a biomarker for the early diagnosis of OSCC [32]. Our in vitro study showed that knockdown of LINC02147 promoted the proliferation of hBMFs and SCC-9 cells and elevated the expression levels of MCM2, MCM3 and MCM5 in hBMFs and SCC-9 cells (Figs. 7 and 8), which validated the prediction of GSEA.

Myofibroblasts are principal cells during wound healing and organ fibrosis that secrete collagen and reorganize the extracellular matrix (ECM) [48]. Persistent activation of myofibroblasts often contributes to OSF [49, 50]. Myofibroblasts are formed by the transdifferentiation of various cells. Local fibroblasts in tissues are the predominant source of myofibroblasts. α-SMA is a typical marker of myofibroblasts [39, 51]. Our in vitro study showed that knockdown of LINC02147 led to upregulation of α-SMA in hBMFs, suggesting that low LINC02147 expression may promote the transdifferentiation of hBMFs into myofibroblasts.

Myofibroblasts contribute to ECM production, which contains COL1α1 and FN1 [39]. Vimentin is involved in cell growth and differentiation and was significantly overexpressed in arecoline-treated fibroblasts and OSF tissues, indicating that vimentin may be involved in the pathogenesis of OSF [52–55]. Therefore, α-SMA, COL1α1, FN1, and vimentin are common fibrosis markers of OSF [12, 39, 56, 57]. Our study found that the knockdown of LINC02147 in hBMFs led to the upregulation of α-SMA, COL1α1, FN1, and vimentin (Fig. 6), suggesting that low LINC02147 expression could promote fibrogenesis.

Moreover, studies have shown that α-SMA-positive fibroblasts (myofibroblasts) may identify OSF with a high risk of malignant transformation [58]. Vimentin expression is significantly enhanced during tumorigenesis [59]. Vimentin is a potential marker of oral malignant transformation [60]. Our study found that the knockdown of LINC02147 in hBMFs led to the upregulation of α-SMA and vimentin (Fig. 6). These results further suggested low LINC02147 expression contributed to OSF malignant progression, possibly by promoting the proliferation and differentiation of hBMFs. The exact mechanism needs further study.

Cox regression analysis validated that LINC02147 was an independent prognostic factor for OSCC and was not affected by clinical factors (Fig. 9). In addition to LINC02147, TNM stage and perineural invasion were also independently related to the OS of OSCC. The independent prognostic factors were used to construct a nomogram. The nomogram combines genetic and clinical information to calculate and predict personalized survival rates of OSCC patients, thus helping physicians make diagnosis and treatment decisions [61]. We developed a LINC02147 signature-based nomogram and confirmed its good accuracy and sensitivity, which may have application prospects (Fig. 10).

Our study identified LINC02147 as a novel prognostic signature. Low LINC02147 expression promoted OSF malignant progression and predicted a poorer prognosis of OSCC. Preliminary mechanistic experiments suggested that LINC02147 may be involved in OSF malignant progression by negatively regulating cell proliferation and the MCM pathway. Although our study produced valuable insights, it still had limitations. First, due to this study’s limited clinical sample size, a more extensive study should be conducted to investigate the characteristics of LINC02147 in the future. Second, the LINC02147 signature-based nomogram can only predict the postoperative survival rate of OSCC but not the cancer risk of OSF. Based on the above considerations, clinicopathological data from more extensive multicentre OSCC patients with OSF will be collected to further confirm the prognostic value of LINC02147 and construct a nomogram model to predict the cancer risk of OSF. Rescue experiments and further mechanistic studies will also be carried out. In addition, the present study did not differentiate OSF with or without dysplasia. Whether the function of LINC02147 differs in OSF with and without dysplasia is worth studying in the future.

## Conclusion

We used bioinformatic methods, clinical tissue samples, and in vitro study to verify that LINC02147 was gradually downregulated from NOM to OSF to OSCC, with the lowest expression levels in OSCC cells and tissues. Moreover, LINC02147 acted as a potential prognostic and diagnostic biomarker for OSCC, and low LINC02147 expression predicted poor prognosis for OSCC, indicating an essential role of LINC02147 during OSF malignant progression. In our future study, the predictive value of the LINC02147 signature-based nomogram must be verified by clinical data, and the inherent mechanism of LINC02147 needs to be unveiled.

## Supplementary Information

**Additional file 1**: **Table S1**. Basic information of two datasets. **Table S2**. Primer sequence for qPCR. **Table S3**. Clinical Characteristics of the 326 OSCC Patients in the TCGA cohort. **Table S4**. Differentially Expressed lncRNAs in the ceRNA networks related to OSF malignant progression.

**Additional file 2**: Supplementary Methods.

**Additional file 3**: Supplementary Results.

**Additional file 4**: **Fig. S1**.The relative expression of RP11-108K3.1 in NOM, OSF and OSCC clinical tissue samples. Expression differences were compared by ordinary one-way ANOVA test. (*P < 0.05, **P < 0.01, ***P < 0.001, ****P < 0.0001).

**Additional file 5**: Supplementary figure 2.

**Additional file 6**: Supplementary figure 3.

## Data Availability

The data generated during this study are openly available in GEO, TCGA, and Molecular Signatures Database at https://www.ncbi.nlm.nih.gov/geo/query/acc.cgi?acc=GSE125866, https://www.ncbi.nlm.nih.gov/geo/query/acc.cgi?acc=GSE64216, https://www.ncbi.nlm.nih.gov/geo/query/acc.cgi, https://portal.gdc.cancer.gov/repository, http://www.broad.mit.edu/gsea/msigdb/index.jsp.

## Abbreviations

OSF: Oral submucous fibrosis
OSCC: Oral squamous cell carcinoma
NOM: Normal oral mucosa
OS: Overall survival
GEO: Gene expression omnibus
TCGA: The cancer genome atlas
ceRNA: Competitive endogenous RNA
DEGs: Differentially expressed genes
DEGA: Differentially expressed gene analysis
WGCNA: Weighted gene co-expression network analysis
GSEA: Gene set enrichment analysis
qPCR: Quantitative polymerase chain reaction
K-M: Kaplan–Meier
AUC: Area under the curve
hBMFs: Human buccal mucosa fibroblasts
α-SMA: α-Smooth muscle actin
COL1α1: Collagen type 1, α1
FN1: Fibronectin
