# Correction to: Low LINC02147 expression promotes the malignant progression of oral submucous fibrosis

**DOI:** 10.1186/s12903-022-02395-9

**Published:** 2022-09-14

**Authors:** Jun Chen, Wenjie Li, Binjie Liu, Xiaoli Xie

**Affiliations:** 1 Hunan Key Laboratory of Oral Health Research & Hunan 3D, Printing Engineering Research Center of Oral Care and Hunan Clinical Research Center of Oral Major Diseases and Oral Health and Xiangya Stomatological Hospital and Xiangya School of Stomatology, Central South University, 72 Xiangya Road, Kaifu District, Changsha, 410008 People’s Republic of China; 2 State Key Laboratory of Powder Metallurgy, Central South University, Changsha, 410083 People’s Republic of China; 3 Department of Oral Health Science, School of Dentistry, University of Washington, Seattle, WA 98195 USA

## Correction to: BMC Oral Health (2022) 22:316 10.1186/s12903-022-02346-4

In the original version of this article, there is a typo in Fig. 1. The number “327” is actually “326”. The corrected Fig. 1 is given below.

**Fig. 1:**
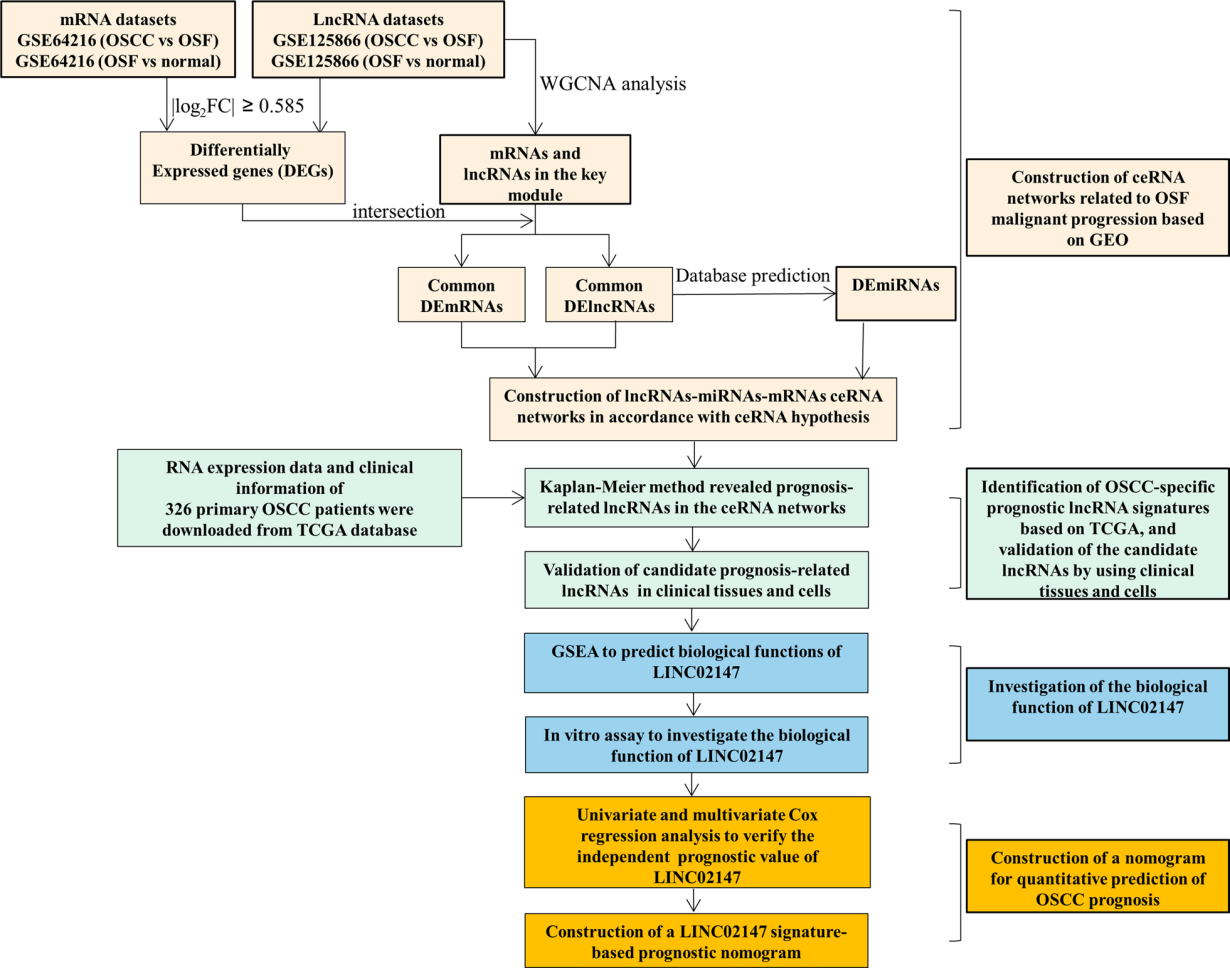
Workflow of this study. DEmRNAs, differentially expressed mRNAs; DElncRNAs, differentially expressed mRNAs; OSF, oral submucous fibrosis; OSCC, oral squamous cell carcinoma; GEO, Gene Expression Omnibus; TCGA, The Cancer Genome Atlas; WGCNA, weighted gene co-expression network analysis; GSEA, gene set enrichment analysis

In the original version of this article, there is a typo in the legend of Fig. 7. “A–B Knockdown of LINC02147 promoted cell proliferation of SCC-9” is actually “A–B CCK-8 assay showed that knockdown of LINC02147 promoted cell proliferation of hBMFs”.

