# Supplementary material for: Low LINC02147 expression promotes the malignant progression of oral submucous fibrosis
Source: BMC Oral Health. 2022 Jul 29;22:316. doi: 10.1186/s12903-022-02346-4 (PMC9338683; doi:10.1186/s12903-022-02346-4)
Supplement: Supplementary file 1 — Additional file 1: Table S1. Basic information of two datasets. Table S2. Primer sequence for qPCR. Table S3. Clinical Characteristics of the 326 OSCC Patients in the TCGA cohort. Table S4. Differentially Expressed lncRNAs in the ceRNA networks related to OSF malignant progression. [file 12903_2022_2346_MOESM1_ESM.docx]

| **Supplementary Table 1. Basic information of two datasets** | | | |
| --- | --- | --- | --- |
| Year | series | Platform | Samples |
| 2019 | GSE125866 | GPL20795 Hiseq X Ten (Homo sapiens) | OSF:OSCC:Normal=8:8:2 |
| 2016 | GSE64216 | GPL10588 Illumina HumanHT-12 V4.0 expression beadchip | OSF:OSCC:Normal=4:2:2 |

Supplementary Table 1. GSE125866 (8 OSF, 8 OSCC and 2 normal oral mucosa tissues) was associated with the GPL20795 (GPL20795 Hiseq X Ten, Homo sapiens).

GSE64216 (4 OSF, 2 OSCC and 2 normal oral mucosa tissue specimens) was associated the GPL10558 platform (Illumina HumanHT-12 V4.0 expression beadchip).

| **Supplementary Table 2. Primer sequence for qPCR** | | |
| --- | --- | --- |
| Gene |  | Sequence (5’-3’) |
| LINC02147 | Forward | GTGCTATCAGGGTTGGTTTCG |
|  | Reverse | CGTGAGGACACTGCGAGAAGAT |
| RP11-108K3.1 | Forward | TCTTGGCTCCTCCTGCTTCT |
|  | Reverse | CCTGCAATCGCAGGCACT |
| α-SMA | Forward | GGGGTGATGGTGGGAATG |
|  | Reverse | AGCAGGGTGGGATGCTCTT |
| COL1α1 | Forward | TCTCCACCTGCCTCTGGC |
|  | Reverse | TTGTGCTTTGGGAAGTTGTCTC |
| Vimentin | Forward | AAATGGCTCGTCACCTTCGT |
|  | Reverse | CAACCAGAGGGAGTGAATCCA |
| FN1 | Forward | CAAGCATCACCCTGGGAGTT |
|  | Reverse | GCATCTTGGTTGGCTGCATA |
| MCM2 | Forward | AAGACTTTTGCCCGCTACCT |
|  | Reverse | ATCTGACGAGCCTTATCCACC |
| MCM3 | Forward | ACCAAGGAATCCCAGAAAGTG |
|  | Reverse | CAGAGCAGCCTGGATCTCAA |
| MCM5 | Forward | CGGCTCTTCCAAGTGTCCA |
|  | Reverse | CGTGCTCCGGGTATTTCTG |
| GAPDH | Forward | CCCACTCCTCCACCTTTGAC |
|  | Reverse | TCTTCCTCTTGTGCTCTTGCTG |
| U6 | Forward | CTCGCTTCGGCAGCACA |
|  | Reverse | AACGCTTCACGAATTTGCGT |

**Supplementary Table 3. Clinical Characteristics of the 326 OSCC Patients in the TCGA cohort**

| **Characteristics** |  | **Number (%)** | **Characteristics** |  | **Number (%)** |
| --- | --- | --- | --- | --- | --- |
| Gender | Male | 224 (68.71) | N Stage | N0 | 166 (50.92) |
|  | Female | 102 (31.29) |  | N1 | 57 (17.48) |
| Age | ≤58 | 127 (38.96) |  | N2 | 10 (3.07) |
|  | ＞58 | 199 (61.04) |  | N2a | 8 (2.45) |
| Tumor Site | Oral tongue | 123 (37.73) |  | N2b | 44 (13.50) |
|  | Base of tongue | 21 (6.44) |  | N2c | 24 (7.36) |
|  | Buccal mucosa | 22 (6.75) |  | N3 | 3 (0.92) |
|  | Floor of mouth | 60 (18.40) |  | NX | 11 (3.37) |
|  | Hard Palate | 7 (2.15) |  | Not available | 3 (0.92) |
|  | Lip | 3 (0.92) | TNM Stage | I | 19 (5.83) |
|  | Oral Cavity | 90 (27.61) |  | II | 53 (16.26) |
| Pathological Grading | G1 | 51 (15.64) |  | III | 60 (18.40) |
|  | G2 | 198 (60.74) |  | IVA | 159 (48.77) |
|  | G3 | 67 (20.56) |  | IVB | 6 (1.84) |
|  | G4 | 2 (0.61) |  | Not available | 29 (8.90) |
|  | GX | 6 (1.84) |  |  |  |
|  | Not available | 2 (0.61) | Lymphovascular Invasion | Yes | 73 (22.39) |
| T Stage | T1 | 20 (6.13) |  | No | 163 (50.00) |
|  | T2 | 103 (31.60) |  | Not available | 90 (27.61) |
|  | T3 | 80 (24.54) | Perineural Invasion | Yes | 133 (40.80) |
|  | T4 | 18 (5.52) |  | No | 115 (35.28) |
|  | T4a | 92 (28.22) |  | Not available | 78 (23.93) |
|  | T4b | 3 (0.92) |  | | |
|  | TX | 7 (2.15) |  |  |  |
|  | Not available | 3 (0.92) |  |  |  |

| **Supplementary Table 4.** Differentially Expressed lncRNAs in the ceRNA networks related to OSF malignant progression | | | | | | |
| --- | --- | --- | --- | --- | --- | --- |
| **LncRNA ID in datasets** | **Ensembl gene ID** | **HGNC Gene Symbol** | **Regulation in datasets** | **Prognostic value (K-M method)** | **Diagnostic value (ROC analysis)** | **Expression in clinical samples** |
| RP11-108K3.1 | ENSG00000259240 | MIR4713HG | up | **+** | **+** | down |
| RP11-203M5.8 | ENSG00000258908 | lnc-PIP4P1-1 | up | - | ※ | ※ |
| AF127577.10 | ENSG00000229047 | lnc-RBM11-3 | up | - | ※ | ※ |
| AC007563.5 | ENSG00000236886 | lnc-IGFBP2-8 | down | - | ※ | ※ |
| RP11-475O6.1 | ENSG00000233008 | LINC01725 | down | **+** | - | ※ |
| RP11-24J23.2 | ENSG00000223949 | ROR1-AS1 | down | - | ※ | ※ |
| RP11-420A23.1 | ENSG00000251432 | lnc-LARP1B-1 | down | - | ※ | ※ |
| CTD-2516F10.2 | ENSG00000251364 | lnc-CYB5R2-3 | down | - | ※ | ※ |
| CTD-3179P9.1 | ENSG00000249797 | LINC02147 | down | **+** | **+** | down |
| RP11-526F3.1 | ENSG00000251076 | lnc-APC-9 | down | - | ※ | ※ |
| RP11-384F7.2 | ENSG00000239268 | lnc-LSAMP-1 | down | - | ※ | ※ |
| “+” means a positive result, “-” means a negative result, “※” means no tests have been done. | | | | | | |
