# Supplementary material for: Low LINC02147 expression promotes the malignant progression of oral submucous fibrosis
Source: BMC Oral Health. 2022 Jul 29;22:316. doi: 10.1186/s12903-022-02346-4 (PMC9338683; doi:10.1186/s12903-022-02346-4)
Supplement: Supplementary file 2 — Additional file 2: Supplementary Methods. [file 12903_2022_2346_MOESM2_ESM.docx]

**Supplementary Methods**

**Kaplan-Meier survival analysis**

To identify lncRNAs with OSCC-specific prognostic characteristics in the ceRNA networks, the RNA-seq data and clinical data of head and neck squamous cell carcinoma (HNSCC) were downloaded from TCGA (http://tcgadata.nci.nih.gov/). Among which, 327 OSCC patients with no history of malignancy or neoadjuvant therapy were included in our study. Kaplan-Meier (K-M) method was used to calculate the correlation between overall survival of OSCC patients and the 11 lncRNAs in the ceRNA networks. According to the gene expression of each sample, the best cutoff value was selected by X-tile software as the threshold value of the high expression group and the low expression group. Patients were classified according to the threshold value. X-tile plots provide an intuitive way to assess the relationship between variables and survival. The X-tile software version 3.6.1 (Yale University School of Medicine, New Haven, CT, USA) was performed to do the X-tile plots. The “survival” (https://cran.r-project.org/web/packages/survival/index.html) in “R” was used to calculate survival rate and “ggplot2” was performed to plot K-M curve. Moreover, in order to further confirm the prognostic roles of the candidate lncRNAs, we analyzed their expression levels in different clinical stages of OSCC patients in TCGA.

**Quantitative PCR (qPCR)**

The expressions of LINC02147, α-SMA, COL1α1, FN1, vimentin, MCM2, MCM3 and MCM5 at the RNA level were examined by qPCR. Total RNA was extracted from tissue samples and cells by using Trizol reagent (Invitrogen, USA). Extracted RNA was reversely transcribed to complementary DNA (cDNA) by using Reverse Transcription Kit (Takara, Tokyo, Japan). The PCR reaction system and reaction conditions were prepared according to the protocol. The relative expression levels of genes were calculated by using the 2^-ΔΔCt^ method. GAPDH was utilized as the housekeeping gene for normalization. The primer sequences are listed in [supplementary Table 3](#supplementaryTabel3).

**Western Blot**

RIPA lysis and extraction buffer (Thermo Scientific, USA) was used to isolate protein extracts from each group cells according to the user guide. Total protein was isolated by 10% SDS-PAGE, transferred by polyvinylidene fluoride membrane, blocked by 5% BSA (Solarbio, Beijing, China), and probed with appropriate primary antibodies to the target proteins. The following antibodies were used: anti-α-SMA (Abcam, ab124964), anti-COL1α1 (Abcam, ab260043), anti-FN1 (Abcam, ab45688), anti-vimentin (Abcam, ab92547) and anti-β-actin (Immunoway, YM3028). Then, samples were subjected to western blot.

**Independent prognostic value analysis**

Univariate Cox proportional hazards regression analysis was used to evaluate the prognostic value of each candidate biomarkers and clinical parameters, and multivariate Cox analysis was used to identify the independent prognostic factors. Thus, to further validate the prognostic association of LINC02147 in OSCC patients, univariate Cox regression analysis was performed by using "survival" package in “R”. Statistical p value < 0.05 was set as the cut-off criteria. The data of 327 OSCC patients were coded and input into a computer to establish a database. The clinical characteristics of the 327 OSCC patients were shown in supplementary Table2. Nine characteristics were selected for univariate Cox regression analysis, including gender, age, pathological grading, lympho-vascular invasion, perineural invasion, T stage, N stage, TNM stage and LINC02147 expression. χ2 test was used for univariate Cox regression analysis. Next, the characteristics with statistical significance in univariate Cox regression analysis were selected for multivariate Cox regression analysis. In Cox regression model, the coefficient of some independent variable is taken as a power of “*e*”, and the value obtained is the value of hazard ratios (HR). When HR>1, the characteristic is considered as a risk factor. When HR<1, the characteristic is considered as a protective factor.
