# Supplementary material for: Low LINC02147 expression promotes the malignant progression of oral submucous fibrosis
Source: BMC Oral Health. 2022 Jul 29;22:316. doi: 10.1186/s12903-022-02346-4 (PMC9338683; doi:10.1186/s12903-022-02346-4)
Supplement: Supplementary file 3 — Additional file 3: Supplementary Results. [file 12903_2022_2346_MOESM3_ESM.docx]

**Supplementary Results**

**Identification of DEGs**

The mRNA expression profiles of 4 OSF samples and 2 NOM samples were compared. [Figure. 1A](#Figure1) showed the volcano map and [Figure. 1E](#Figure1) showed the heat map. A total of 1963 DEmRNAs were significantly upregulated, and 2712 were downregulated in OSF tissues compared with NOM. The mRNA expression profiles of 2 OSCC and 4 OSF samples were compared. [Figure. 1B](#Figure1) showed the volcano map and [Figure. 1F](#Figure1) showed the heat map. A total of 2755 DEmRNAs were significantly upregulated, and 2366 were downregulated in OSCC tissues compared with OSF. 271 DEmRNAs with a consistently sequential change from NOM to OSF to OSCC were identified, among which 93 were consistently upregulated ([Figure. 1I](#Figure1)) and 178 were consistently downregulated ([Figure. 1J](#Figure1)).

The lncRNA expression profiles of 8 OSF and 2 NOM samples were compared. [Figure. 1C](#Figure1) showed the volcano map and [Figure. 1G](#Figure1) showed the heat map. A total of 93 DElncRNAs were significantly upregulated, and 162 were downregulated in OSF tissues compared with NOM. The lncRNA expression profiles of 8 OSCC and 8 OSF samples were compared. [Figure. 1D](#Figure1) showed the volcano map and [Figure. 1H](#Figure1) showed the heat map. A total of 293 DElncRNAs were significantly upregulated, and 166 were downregulated in OSCC tissues compared with OSF. 21 DElncRNAs with a consistently sequential change from NOM to OSF to OSCC were identified, among which 8 were consistently upregulated ([Figure. 1K](#Figure1)) and 13 were consistently downregulated ([Figure. 1L](#Figure1)).


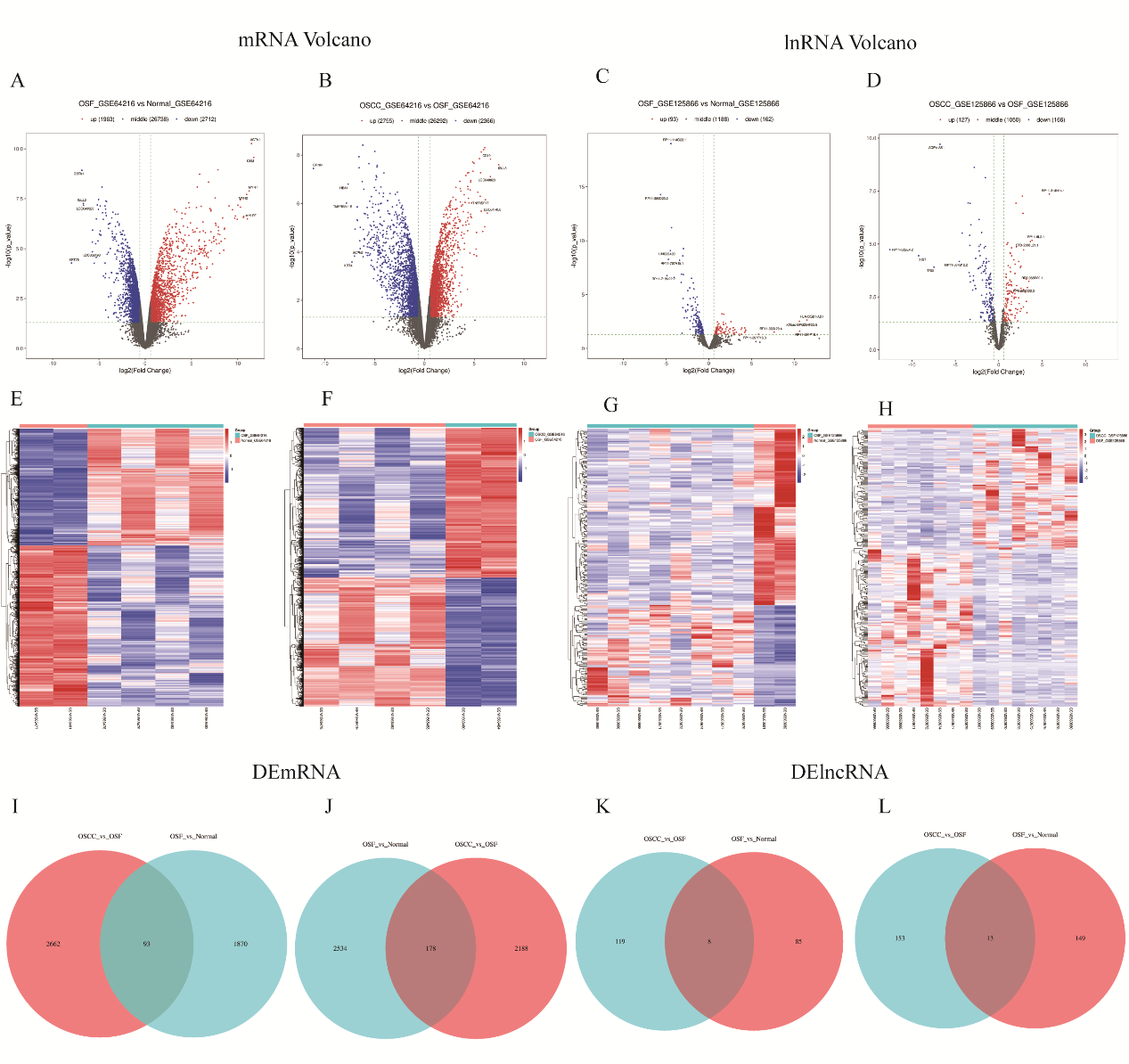


**Figure 1. Differentially expressed genes analysis (DEGA) in OSF malignant transformation.** (A) Volcano plot of DEmRNAs between OSF and normal oral mucosa (NOM). (B) Volcano plot of DEmRNAs between OSF and OSCC. (C) Volcano plot of DElncRNAs between OSF and NOM. (D) Volcano plot of DElncRNAs between OSF and OSCC. (E) The hierarchial clustering heat map of DEmRNAs between OSF and NOM. (F) The hierarchial clustering heat map of DEmRNAs between OSF and OSCC. (G) The hierarchial clustering heat map of DElncRNAs between OSF and NOM. (H) The hierarchial clustering heat map of DElncRNAs between OSF and OSCC. (I) Venn diagram of up-regulated DEmRNAs in the progression of OSF. (J) Venn diagram of down-regulated DEmRNAs in the progression of OSF. (K) Venn diagram of up-regulated DElncRNAs in the progression of OSF. (L) Venn diagram of down-regulated DElncRNAs in the progression of OSF.

**Identification of co‑expression module by WGCNA**

The sample clustering dendrograms of the NOM, OSF and OSCC conditions are shown in [Figure. 2A](#Figure2). We constructed the weighted adjacency matrix by using a power function based on a soft-threshold parameter β. In this study, we selected β=10 (scale free R^2^=0.829) as the soft threshold power to ensure a scale-free network ([Figure. 2B](#Figure2)). A total of 19 modules were identified ([Figure. 2C~D](#Figure2)). The correlation between each co-expression module and the grouping (NOM=0, OSF=1, OSCC=2) was calculated. The modules with the most significant correlation were selected, among which “Brown” had the most significant positive correlation and “Orangered4 + PLUM1” had the most significant negative correlation.


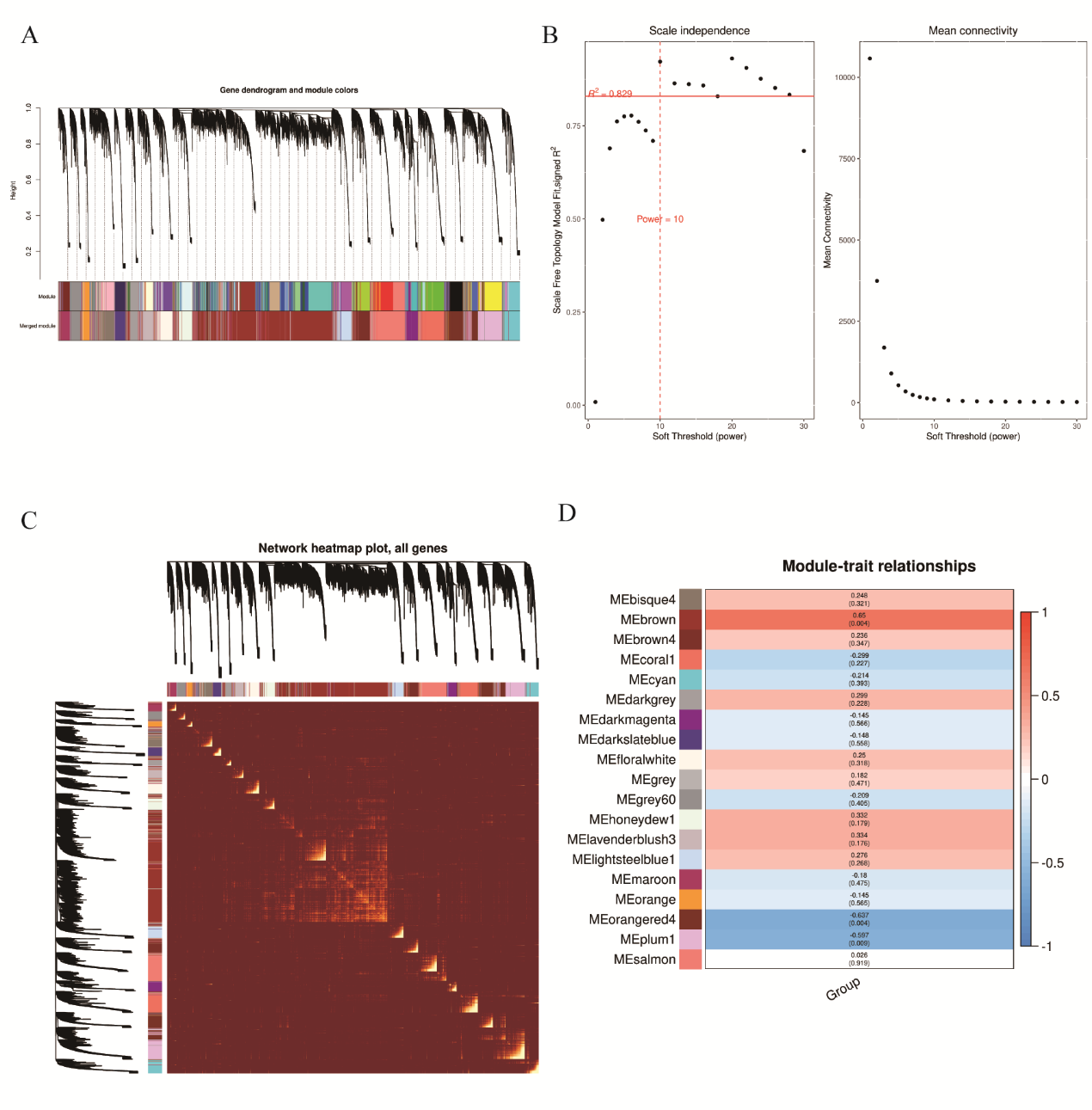


**Figure 2. Co-expression network analysis by WGNCA.** (A) Dendrogram of all differentially expressed genes clustered based on a dissimilarity measure (1-TOM). (B) Analysis of the scale-free fit index for various soft-threshold powers and analysis of the mean connectivity for various soft-threshold powers. (C) Network heat map for all genes. (D) The relationships between modules and clinical traits.

**Biological function and pathway enrichments of DEGs in the ceRNA networks**

GO function and KEGG enrichment analyses of DEGs in the ceRNA network were performed using the clusterProfiler package in R. Under the threshold＜0.05, biological processes of the mRNAs were suggested to focus on formation of primary germ layer (p=2.17e-05), positive regulation of transcription, DNA templated (2.49e-05), positive regulation of transcription by RNA polymerase II (p=3.08e-05), outflow tract morphogenesis (p=3.90e-05), positive regulation of nucleic acid-templated transcription(6.16e-05), positive regulation of RNA biosynthetic process (p=6.21e-05), positive regulation of cellular biosynthetic process (p=0.000123), positive regulation of mitotic cell cycle (p=0.000123), and positive regulation of cell cycle(p=0.000129) ([Fig.3A~B](#Figure3)).

While KEGG pathway enrichment analysis suggested that the DEGs in the ceRNA network was significantly enriched in endocrine resistance (p= 0.000296), mucin type O-glycan biosynthesis (p=0.000887), transcriptional misregulation in cancer (p=0.00560), prolactin signaling pathway (p=0.009127), p53 signaling pathway (p=0.009858), signaling pathways regulating pluripotency of stem cells (p= 0.010733), longevity regulating pathway (p=0.017449), Th1 and Th2 cell differentiation (p=0.0190489), inflammatory mediator regulation of TRP channels (p= 0.023701), Toll-like receptor signaling pathway (p=0.02623), and TNF signaling pathway (p=0.03173) ([Fig.3C~D](#Figure3)).

The results showed that significant GO terms in BP and MF categories were mainly associated with cell proliferation, suggesting abnormal cell proliferation may be involved in OSF canceration. KEGG pathway analysis indicated that mucin type O-glycan biosynthesis, transcriptional misregulation in cancer, p53 signaling pathway, signaling pathways regulating pluripotency of stem cells, longevity regulating pathway, Th1 and Th2 cell differentiation, inflammatory mediator regulation of TRP channels, Toll-like receptor signaling pathway and TNF signaling pathway were enriched. These pathways were mainly involved in proliferation, differentiation and inflammation, indicating that lncRNA-mediated abnormal cell proliferation, immunity and inflammation take part in the progression of OSF to OSCC.


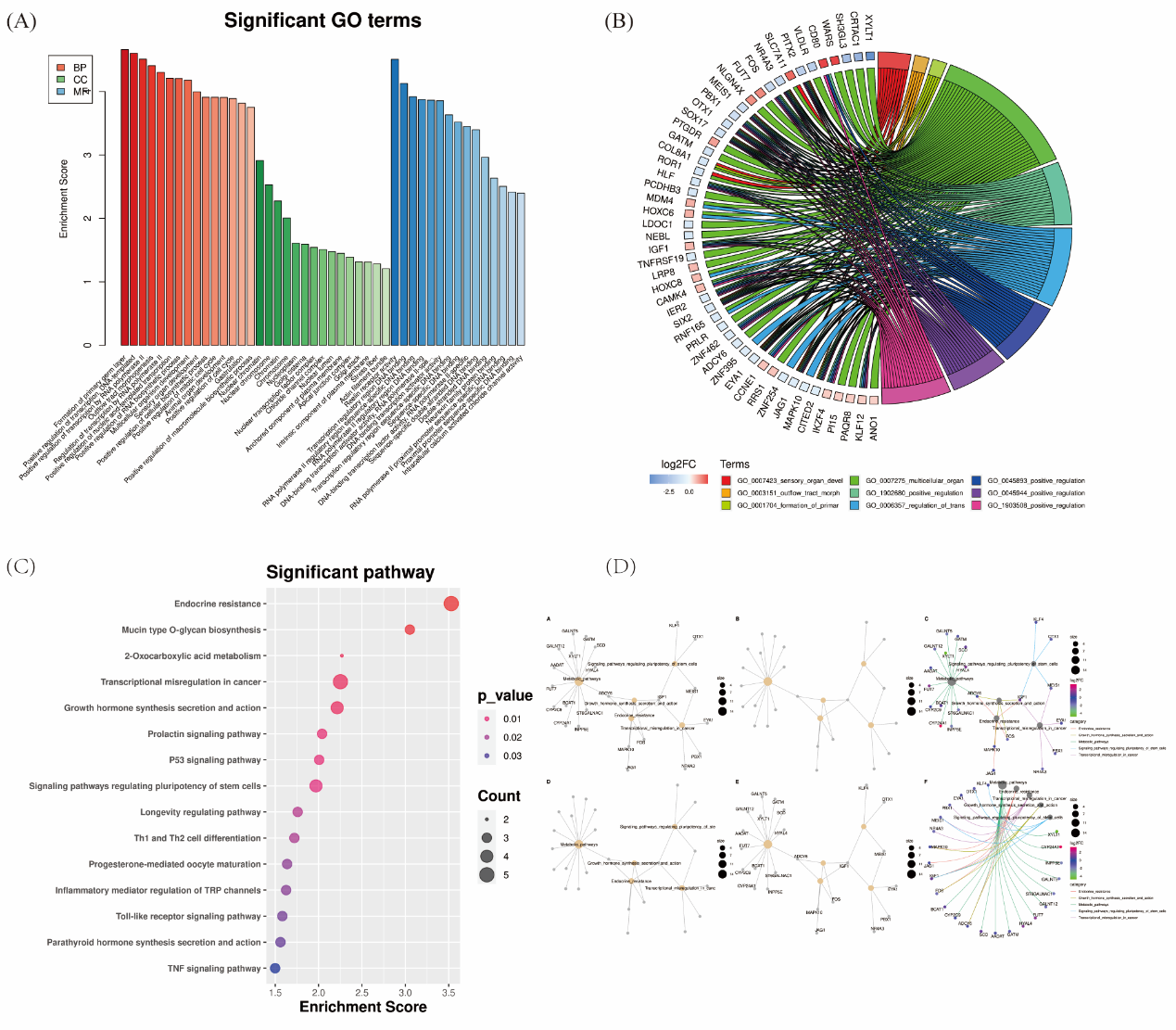


**Figure 3. Go function enrichment and KEGG analysis of DEGs associated with the malignant progression of OSF to OSCC.** (A) The ESbar shows the top 15 GO terms to affect biological process (BP), molecular function (MF) and cellular component (CC). (B) The circle shows the correlation between statistically top 50 DEmRNAs and their GO terms. (C) The dotplot shows the top 15 pathways of lncRNA-related DEmRNAs. (D) 5 pathways were used Cytoscape for constructing a pathway-gene network with DEmRNAs.
