# Supplementary material for: Low LINC02147 expression promotes the malignant progression of oral submucous fibrosis
Source: BMC Oral Health. 2022 Jul 29;22:316. doi: 10.1186/s12903-022-02346-4 (PMC9338683; doi:10.1186/s12903-022-02346-4)
Supplement: Supplementary file 4 — Additional file 4: Fig. S1.The relative expression of RP11-108K3.1 in NOM, OSF and OSCC clinical tissue samples. Expression differences were compared by ordinary one-way ANOVA test. (*P < 0.05, **P < 0.01, ***P < 0.001, ****P < 0.0001). [file 12903_2022_2346_MOESM4_ESM.docx]

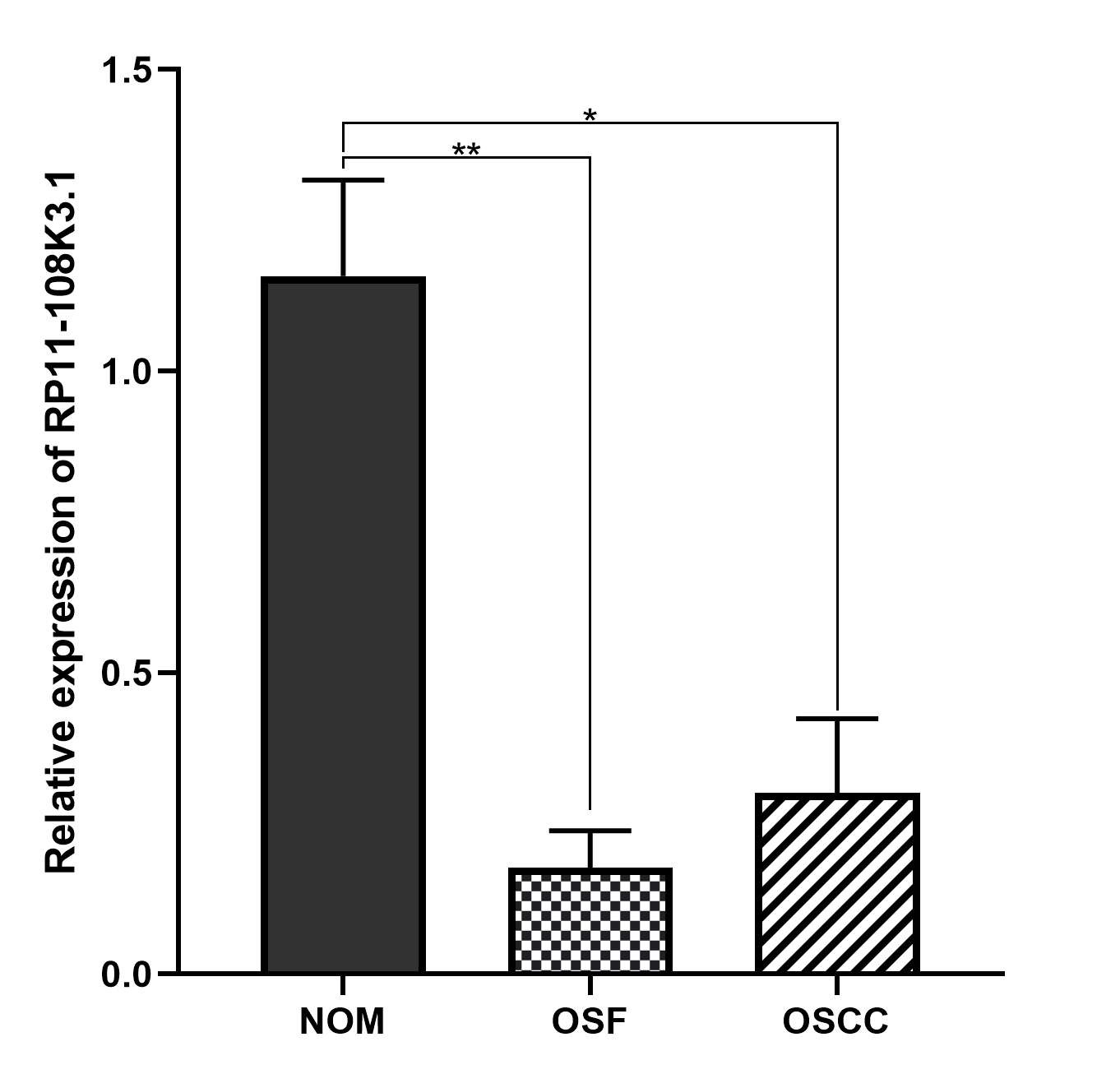


**Supplementary Figure 1.** The relative expression of RP11-108K3.1 in NOM, OSF and OSCC clinical tissue samples. Expression differences were compared by ordinary one-way ANOVA test. (*P < 0.05, **P < 0.01, ***P < 0.001, ****P < 0.0001).
