# Supplementary figures and images for: Low LINC02147 expression promotes the malignant progression of oral submucous fibrosis
Source: BMC Oral Health. 2022 Jul 29;22:316. doi: 10.1186/s12903-022-02346-4 (PMC9338683; doi:10.1186/s12903-022-02346-4)

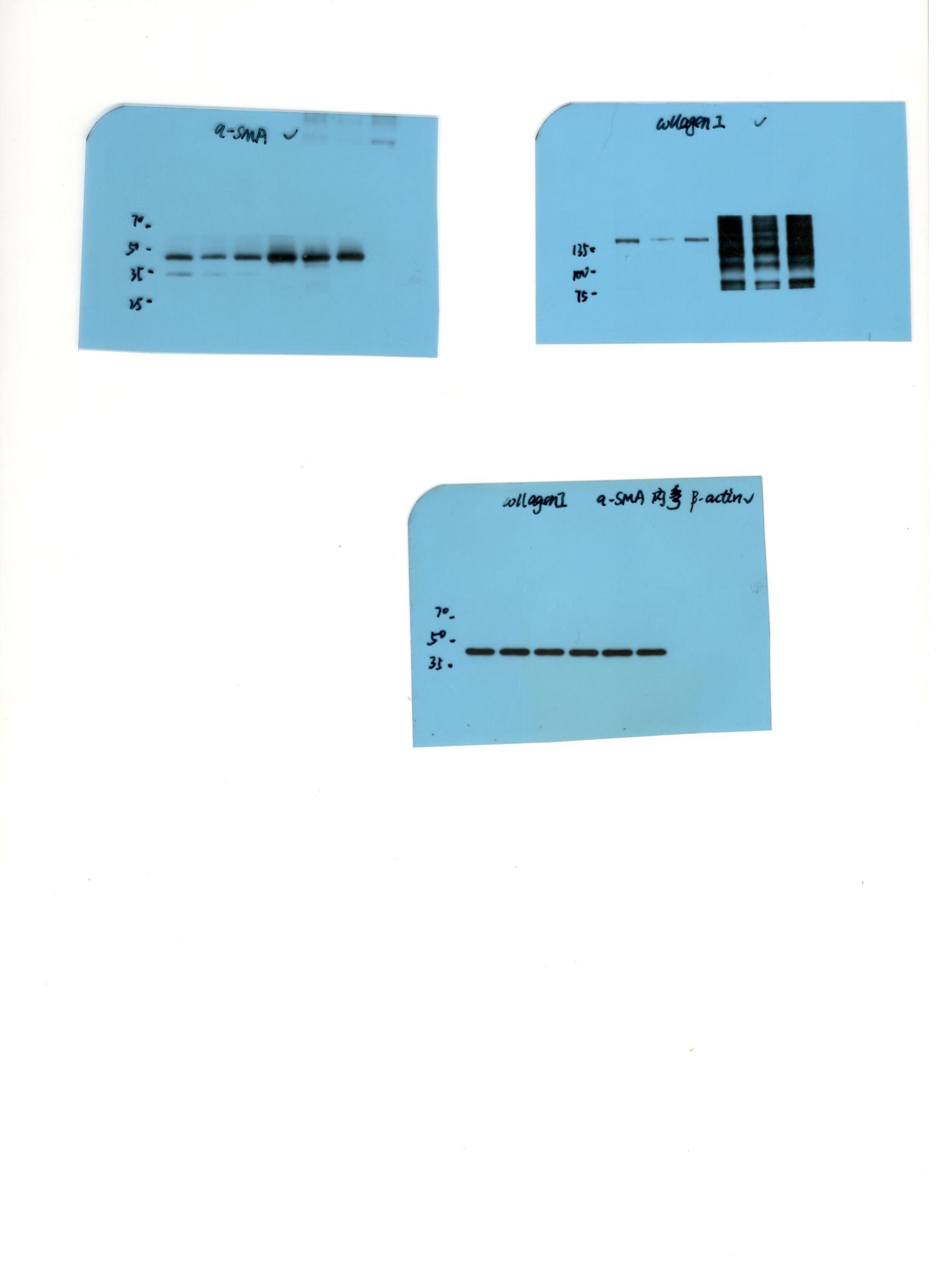

Supplement: Supplementary file 5 — Additional file 5: Supplementary figure 2. [file 12903_2022_2346_MOESM5_ESM.jpg]

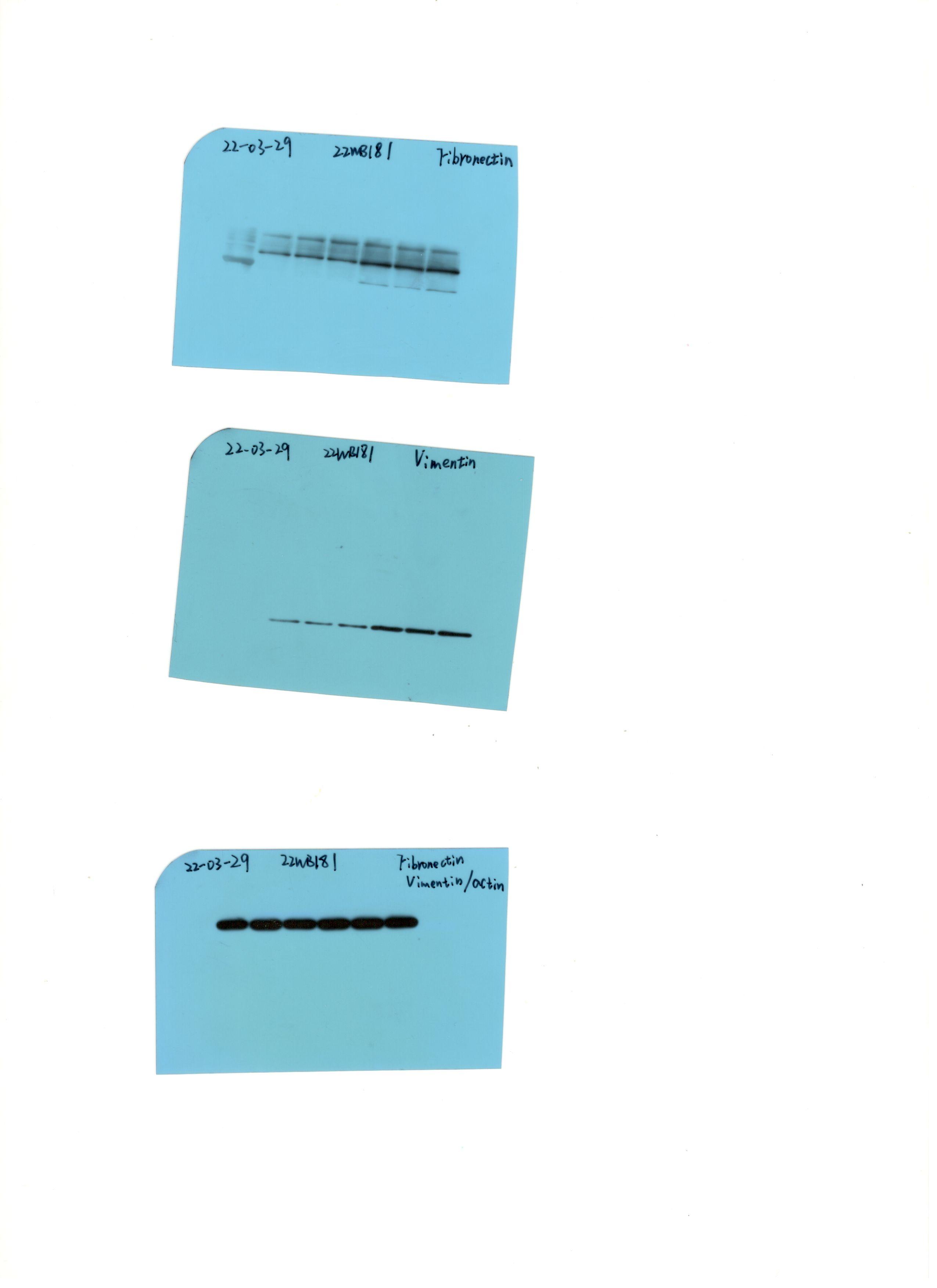

Supplement: Supplementary file 6 — Additional file 6: Supplementary figure 3. [file 12903_2022_2346_MOESM6_ESM.jpg]
